# Metabolomic and Proteomic Profiling of Athletes Performing Physical Activity under Hypoxic Conditions

**DOI:** 10.3390/sports12030072

**Published:** 2024-03-05

**Authors:** Kristina A. Malsagova, Arthur T. Kopylov, Alexander A. Stepanov, Liudmila I. Kulikova, Alexander A. Izotov, Ksenia A. Yurku, Evgenii I. Balakin, Vasiliy I. Pustovoyt, Anna L. Kaysheva

**Affiliations:** 1 Institute of Biomedical Chemistry, 119121 Moscow, Russia; a.t.kopylov@gmail.com (A.T.K.); aleks.a.stepanov@gmail.com (A.A.S.); likulikova@mail.ru (L.I.K.); izotov.alexander@gmail.com (A.A.I.); kaysheva1@gmail.com (A.L.K.); 2 Institute of Mathematical Problems of Biology RAS—The Branch of Keldysh Institute of Applied Mathematics of Russian Academy of Sciences, 142290 Pushchino, Russia; 3 State Research Center—Burnasyan Federal Medical Biophysical Center of Federal Medical Biological Agency, 119435 Moscow, Russia; ks_yurku@mail.ru (K.A.Y.); vipust@yandex.com (V.I.P.)

**Keywords:** metabolome, proteome, molecular profile, athletes, mass spectrometry analysis

## Abstract

Proteomic and metabolomic research enables quantitation of the molecular profile of athletes. Multiomic profiling was conducted using plasma samples collected from 18 male athletes performing aerobic activity (running) at high altitude. Metabolomic profiling detected changes in the levels of 4-hydroxyproline, methionine, oxaloacetate, and tyrosine during the recovery period. Furthermore, proteomic profiling revealed changes in expression of proteins contributing to the function of the immune system, muscle damage, metabolic fitness and performance, as well as hemostasis. Further research should focus on developing metabolic models to monitor training intensity and athlete adaptation.

## 1. Introduction

Physical activity, amateur, and/or high-performance sports are complex multifactorial processes involving the biological (interactions of genes, proteins, metabolites, and other molecules), physiological, psychological (motivation), and environmental components. Training affects the athlete’s molecular profile, having an impact on gene expression, epigenetic processes, protein synthesis, energy metabolism, and the metabolome related to it [1].

Today, the analytical methods used to quantify gene expression as well as the levels of proteins, lipids, and metabolites in different organs and tissues are available for identifying different biological molecules. This multiomic profiling is also employed in sports and allows one to identify biomarkers of stress, recovery, and/or physiological adaptation [2,3]. Thus, the “sports phenotype” depends on a combination of different features and characteristics, and multi-profile omics analysis aiming to detect biomarkers characterized by an adequate predictive power and statistical reliability needs to be performed to study it [3,4,5]. Sports genomics makes it possible to identify talent for sports, optimize and improve sport results, as well as predict the risk of sports injuries and the recovery period [6]. Physical training also affects the proteomic composition of the athlete’s body [7]. Plasma proteins are a promising source of information for studying physical exercise, since their levels represent the systemic physiological state of a person [8]; levels of circulating proteins respond to physical exercise [9,10]. Numerous studies have demonstrated that the proteomic composition of blood plasma changes significantly during high-intensity physical training [2,11,12]. Thus, Michael Y. Mi et al. [13] identified the peak protein levels under physical load (brain-derived neurotrophic factor, angiopoietin-like 4, WNT signaling modulators, interleukins, apolipoprotein A1, dystroglycan 1, testican-2, kallikrein 8, etc.). A pool of proteins whose levels changed significantly 1 h after training compared to the baseline level was detected. The levels of such proteins as phospholipase A2 receptor, transmembrane glycoprotein, and fibrinogen-like protein 1 were significantly reduced. It was demonstrated previously that physical training increased renal protein clearance 40-fold [14]; this fact can explain why a decrease in plasma protein level is observed after physical training.

The variations in protein expression levels detected when studying different samples over time at different training stages of microcycles and mesocycles as well as during the recovery period allow one to analyze the individual metabolic pathways involved in adaptation to physical load in athletes.

Protein degradation fragments can act as indicators of normal or pathological processes, which can be used for detecting novel markers or mediators of different body states. Extracellular matrix remodeling, when the damaged proteins are degraded and replaced by new ones, is the key process in tissue homeostasis. Cell–matrix interaction during tissue remodeling is preceded by specific proteolytic activity. Extracellular matrix remodeling is disturbed in the pathological state, which involves inflammation and fibrosis [15]. Such an unbalanced extracellular matrix remodeling increases the release of metabolic products (neoepitopes (a class of peptides bound to the major histocompatibility complex)) into the systemic blood flow. Tissue damage is accompanied by degradation of endothelial and epithelial cells, which promotes the supply of inflammatory cells responsible for remodeling of the interstitial membrane. Fragments of both the basement membrane and the interstitial matrix formed during this process can potentially play a signaling role, thus stimulating wound healing or fibrosis initiation. Type IV collagen, laminin, and nidogen are the main components of the basement membrane matrix, whereas the interstitial matrix predominantly consists of type I and III collagen [16].

Contemporary metabolomics enables identification of novel predictors and biomarkers in the field of sports medicine, including for creating personalized recommendations for athletes’ workout sessions [17]. Metabolites are end products of complex interactions occurring inside the cell (at the genomic, transcriptomic, and proteomic levels) and during extracellular events (the environment). The interplay between the genes and the environment can be assessed by comprehensive analysis of the metabolite composition.

Genome-wide association studies enable detection of proteins (a quantitative signature) involved in regulation of a certain metabolic pathway [18]. Identification of novel quantitative signatures in athletes exposed to external factors (diets and high-intensity training) is required for discovering new biomarkers associated with physical exercise and performance.

Thus, elevation in the tyrosine level can be associated with energy requirements (fueling the TCA cycle). In addition, tyrosine can also be an indicator of exercise-induced adaptation and increased physical activity [19].

Due to the availability of blood samples for different tests, plasma or serum samples are ideal candidates for biomarker identification. Other bodily fluids such as cerebrospinal or synovial fluids as well as tissue bioptates may contain other proteins; therefore, investigation of these fluids is more illustrative in some aspects, but sample collection manipulations are associated with greater limitations: invasiveness, risk of injuries, and other undesirable sequelae. Urine is a biological material collected non-invasively but is the least illustrative. As an alternative bodily fluid, sweat also has a number of limitations (dependence of sweat composition on environmental factors, small volume, etc.) [20]. Collecting saliva samples is a non-invasive procedure and causes less discomfort in patients; however, protein concentration in saliva can be 1000-fold lower than that in plasma, thus leading to technological difficulties for diagnosis [21]. Hence, blood derivatives are also preferred because the dynamic range of the content of plasma proteins is more than 11 orders of magnitude [22].

The advances in proteomics and metabolomics have offered a unique opportunity to supplement the recently obtained genomic and transcriptomic data [23]. In sports science, proteomics and metabolomics enable quantitation of proteins and metabolites in professional athletes to identify selective biomarkers of their health [24]. Proteomic and metabolomic studies have identified a number of functional proteins and metabolic pathways varying for different types of physical loads and different athletic disciplines. A number of candidate biomarkers differentiating the functioning of skeletal and cardiac muscles have been identified [25]. In addition to energy processes (glycolysis, lipolysis, and amino acid catabolism), metabolomic studies of professional athletes have identified specific metabolic signatures related to steroid biosynthesis and glutathione metabolism [25]. These signatures can serve as candidate biomarkers of grueling training that professional athletes undertake to improve exercise performance, prevent fatigue-related injuries, and enhance overall health and performance. Changes in these metabolic pathways can also become priceless tools for anti-doping research related to the Athlete Biological Passport, either in combination with other tests or as a standalone test.

The omics data can be used to elaborate optimal personalized training programs for athletes [26]. The results of multiomics research can also be employed to assess fatigue and fitness for training. However, a large sample size is needed for integrating the data from multiple omics approaches and large datasets are obtained, requiring bioinformatics expertise. For this work, there should be collaborative efforts of several research teams that employ common procedures and experimental protocols for conducting multicenter studies aiming to assess physical activity and general lifestyle in order to collect the necessary functional and molecular data to further elucidate the mechanisms responsible for the adaptive response to different training regimens. Such an approach is already being implemented by the Molecular Transducers of Physical Activity Consortium (MoTrPAC) [27]. Hence, the knowledge gained from these large-scale projects will provide information to researchers and health professionals that they can use to develop personalized training protocols aiming to maximize athletes’ performance based on the identified unique molecular signatures.

Furthermore, for any procedure for athlete selection and orientation, there is its own optimal time for application as part of long-term athletic development (Table 1).

Many types of sports induce hypoxia in athletes; some of them are accompanied by significant hypoxemia in the body, with oxygen levels being replenished only during the recovery period.

Exercise-induced hypoxia is accompanied by a number of characteristic signs: hypoxemia and oxygen “debt”, decreased oxygen tension in muscle tissue and mixed venous oxygen saturation, accumulation of underoxidized metabolic products in the blood, pH shift and acid–base imbalance, or increased excessive carbon dioxide production together with oxygen consumption rate in tissues increasing manifold. Exercise-induced hypoxia results from a discrepancy between the increasing cellular oxygen demand and the current oxygen consumption by working muscles. The hypoxic state primarily manifests itself at the cellular level and is inevitable at the beginning of any muscular work, as well as when power output suddenly increases during any athletic exercise, which is accompanied by rising oxygen demand and a higher rate of oxygen supply to working muscles.

It is known that altitude training can improve convective oxygen transport and fitness in athletes [29]. In combination with physical load, hypoxia induces specific responses not observed after identical normoxic training [30]. Hypoxic training is also used to increase the overall metabolic stress, thus providing benefits beyond those achievable under normoxic training conditions [30].

In this study, we assessed the changes in the content of biomolecules in athletes’ plasma at several time points (sampling times) at the metabolomic and proteomic levels caused by physical activity. The main changes were detected at the level of the immune system, muscle damage, hemostasis, metabolic fitness, and performance.

## 2. Materials and Methods

### 2.1. Study Participants

Eighteen male athletes performing aerobic exercise (running) at high altitude (h = 3400 m) were included in the study. The mean age of the study participants was 30.67 ± 4.69 years; BMI was 25.13 ± 1.97 kg/m^2^. 

Table 2 lists the anthropometric characteristics of each study participant.

**Figure 1:**
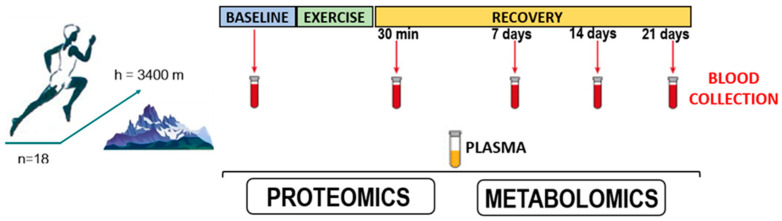
Conditions for collecting biological samples from athletes participating in the study.

Exercise duration was 6 h. Plasma samples were collected at five time points (Figure 1):

The functional characteristics of the study participants are presented in Table 3.

The inclusion criteria were as follows:

- Professional climbers with experience in climbing seven thousand meters and above;
- Age from 24 to 40 years old;
- Male gender;
- Members of the Russian triathlon team;
- Study participants who do not live at high altitudes or have no experience at high altitudes;
- A minimum of 15 years of sports experience;
- Availability of access to training and competitive activities according to an in-depth medical examination.

The exclusion criteria were as follows:

- Females;
- Age below 20 and above 40 years;
- Acute disease and exacerbation of chronic disease at the time of examination;
- Contraindications to stress testing under hypoxic environmental conditions;
- The use of certain pharmacological drugs whose effects may be altered at altitude;
- Lack of willingness of the athlete to comply with study protocols and provide the necessary data.

All the participants were informed of the risks and discomforts associated with the investigation and signed a written consent form to participate. The study was approved by the Board for Ethical Questions at the A.I. Burnazyan State Research Center of the Federal Medical Biological Agency of Russia (Protocol No. 40 dated 18 November 2020).

### 2.2. Training Load Conditions

Training was performed in the morning 1.5 h after breakfast. The study was conducted during summer at an air temperature of 12–18 °C. The heart rate was controlled using a Polar H10 chest strap heart rate monitor (Polar Electro, Kempele, Finland). The athletes were performing exercise in the aerobic heart rate zone (70–80% of the maximum heart rate) along the entire distance (10 km). The mean speed of all the athletes was 16.25 ± 3.12 km/hr. The difference in altitude along the entire distance was <80 m.

### 2.3. Blood Sample Collection

Blood samples were collected from study participants strictly after fasting using the routine procedure from 8 a.m. to 10 a.m. in the clinical diagnostic laboratory of the A.I. Burnazyan State Research Center, Federal Medical Biological Agency of Russia.

For metabolomic and proteomic analyses, blood samples were collected from the cubital vein in vacutainer tubes containing 3.8% sodium citrate anticoagulant (IMPROVACUTER, Guangzhou Improve Medical Instruments Co., Ltd., Guangzhou, China). The samples were centrifuged at 3000 rpm for 6 min at room temperature. Each plasma sample (500 μL) was finally collected in two dry Eppendorf-type polypropylene test tubes, frozen, and stored at −80 °C prior to the analysis. 

Vacutainer tubes containing K_2_EDTA as an anticoagulant were used for the blood biochemistry test. The biomaterial in the vacutainer was centrifuged at 3500 rpm; the supernatant was then transferred into preliminarily labeled polypropylene tubes. For quantitative analysis, plasma samples were frozen at a temperature ≤ −20 °C.

For the hematology test, we used a capillary blood collection system, with three 200 µL capillaries and the following anticoagulants: lithium heparin and ethylenediaminetetraacetic acid. The first drop of blood was discarded into a special biocontainer, and the remaining biomaterial was centrifuged at 3500× *g* no later than 2 h after blood sampling.

### 2.4. Preliminary Preparation of Blood Plasma for HPLC-MS/MS Analysis

Preliminary preparation of blood plasma for proteomic analysis was thoroughly described in [31]. Briefly, the protein fraction was precipitated with methanol (JT Baker, Landsmeer, the Netherlands). Alkylation was performed in the presence of 2% 4-vinylpyridine solution (Aldrich, Gillingham, UK) in 30% isopropanol solution (Fisher Chemical, Laughborough, UK). Trypsinolysis (~800 ng of trypsin/sample, Promega, Madison, WI, USA) involved two stages and was conducted in 75 mM solution of triethylammonium bicarbonate (pH 8.2). To be stored, the tryptic mixtures were vacuum dried (Concentrator Plus, Eppendorf, Hamburg, Germany). Before mass spectroscopy measurements, dry residue was redissolved with 0.1% formic acid (Acros Organics, Geel, Belgium).

The procedure for preliminary plasma preparation for metabolomic analysis was thoroughly described in [31]. Qualitative analysis of 40 endogenic metabolites was performed. The A9906 amino acid standard was used (Sigma, Saint Louis, MO, USA).

### 2.5. Mass Spectrometric Analysis

#### 2.5.1. Proteomic Analysis

Analysis was performed on a Xevo™ G2-XS Q-tof quadrupole time-of-flight mass spectrometer (Waters, Wilmslow, UK) coupled to an Acquity™ UPLC H Class Plus chromatography system (Waters, Wilmslow, UK). The analysis was carried out in the mode of positive electrostatic ionization in the high-sensitivity regimen of the mass analyzer with the normal dynamic range of mass registration. The emitter voltage was 3 kV, the drying gas velocity was 680 L/min, the focusing gas velocity was 50 L/min, the ionization source temperature was 150 °C, and the desolvation temperature was 350 °C. The focusing cone voltage was 67 V with a bias of up to 130 V. The ions were recorded in the hybrid information-independent (DIA) MSE-SONAR mode. The primary information-independent (DIA) MS scan was obtained in the range of 100–1500 *m*/*z*, followed by scanning in the SONAR mode with quadrupole mass isolation in the range of 400 *m*/*z* to 1100 *m*/*z* with an isolation width of 22 Da. The total time for one complete scan cycle was set at 0.418 s. Fragmentation was performed in the two-phase mode: phase 1—low-energy CID fragmentation with argon at 6 eV; phase 2—high-energy graded CID fragmentation with argon in the range of 15 to 37 eV. During the assay, active mass correction *m*/*z* = 556.27 with low activation energy (9 eV) according to the leucine–enkephalin standard (50 pg/mL in 50% acetonitrile with 0.1% formic acid) was used with injection at a speed of 5 µL/min for 30 ms into the ionization source at intervals of 45 s and with isolation within 200 mDa.

Chromatographic separation was performed on an Acquity™ UPLC BEHC18 column (1.7 µm particle size, geometry 2.1 × 50 mm; Waters, Wilmslow, UK) at a flow rate of 0.2–0.3 mL/min and constant incubation of phase A (aqueous solution of 0.1% formic acid and 0.03% acetic acid) and phase B (solution of 0.1% formic acid and 0.03% acetic acid in acetonitrile) at 40 °C with the following elution scheme: 0–1.5 min—3% B, up to 26.5 min—19% B, up to 42 min—32% B, and up to 43.5 min—97% B, which was held in the isocratic mode up to 47.5 min with a decrease of up to 3% B until the 49th minute and then held in the isocratic mode up to 53 min. The flow rate in the interval of 43.5–47.5 min was 0.3 mL/min; in all the other cases it was 0.2 mL/min.

The data obtained were analyzed in the PLGS (Protein Lynx Global Server, version 3.0.3, Waters, Wilmslow, UK) software using the UniProt KB database (version dated March 2021) with preset parameters for the SONAR/MSE scanning mode and preset correction for the calibration mass [31].

#### 2.5.2. Metabolomic Analysis

Data acquisition was performed using a high-resolution quadrupole time-of-flight (Q-TOF) Xevo G2-XS mass spectrometer (Waters, Inc., Wilmslow, UK) equipped with a Z-spray ionization source coupled with a UPLC Acquity H Class system (Waters, Inc., Wilmslow, UK). Details of instrumental analysis are available in [31].

### 2.6. Blood Chemistry and Hematology Tests 

The tests were conducted using a Cobas 6000 modular platform (Roche Diagnostics, Darmstadt, Germany). Table 4 describes the serum biomarkers.

### 2.7. Statistical Analysis 

For more stringent analysis, we narrowed the original proteomics dataset illustrated in the UpSet plot using the R package UpSetR (version 1.4.0) [32] with a sequence of the following steps: (a) Missing quantitative values for the identified protein were imputed with the minimal value found for this protein, (b) proteins that were detected in less than three patients at each time point were removed, (c) proteins that were detected in less than two time points for each patient were removed, and, (d) finally, the missing values of the protein matrix were imputed with the minimal value for the corresponding protein. Protein fold change (FC) was calculated as the median ratio between the protein level (ng on column) after physical load (stress) and the baseline. Proteins for which the ratio was between the maximal and minimal median values in a time series were regarded as differentially expressed proteins (DEPs). 

For the metabolomics data, as well as blood biochemistry and hematology test data, the *p*-value between groups was calculated by pairwise *t*-test followed by Bonferroni correction using the rstatix R package (version 0.7.2). Adjusted *p*-values < 0.05 were regarded as significant.

## 3. Results

### 3.1. Proteome Analysis

**Figure 2:**
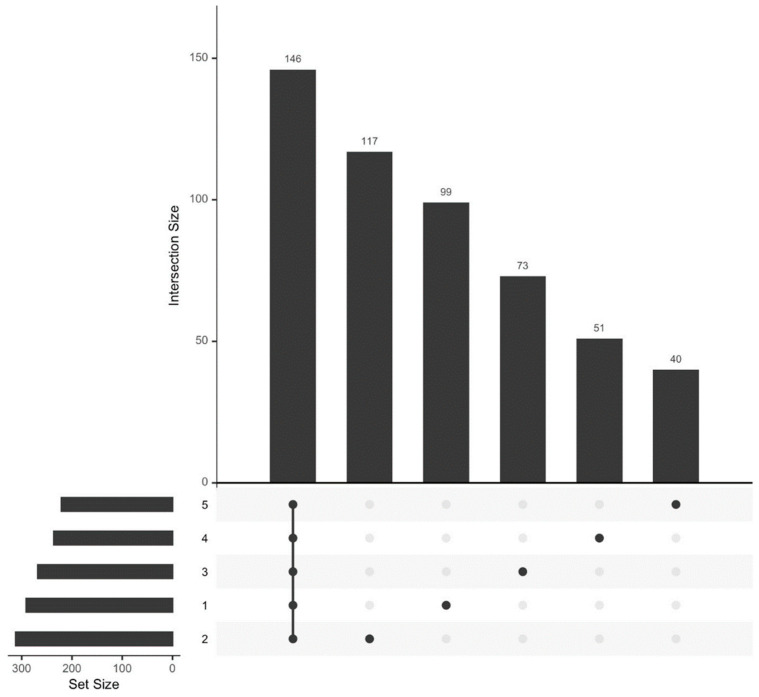
Size of the proteome of the study participants and the number of proteins specific to each biomaterial sampling time.

We analyzed the HPLC-MS/MS data obtained for plasma samples from 18 athletes at five time points (see Section 2.1. Study Participants). Proteins common to all five observation time points comprised a significant fraction of the total proteome (*n* = 146). Ninety-nine unique proteins were identified before exercise (sampling time 1), 117 proteins were identified 30 min after exercise (sampling time 2), 73 proteins were identified 7 days after exercise (sampling time 3), 51 proteins were identified 14 days after exercise (sampling time 4), and 40 proteins were identified 21 days after exercise (sampling time 5) (Figure 2).

Among the identified proteins common for all sampling times, we eventually selected 16 proteins, whose changes in level differed statistically significantly with respect to the first observation time point (i.e., before exercise) (Table 5).

Among the differentially expressed proteins, different isoforms of immunoglobulin light and heavy chains constituted a large group. One can see in Table 5 that there are multi-directional dynamics of protein level variation in the analyzed biological samples. 

**Figure 3:**
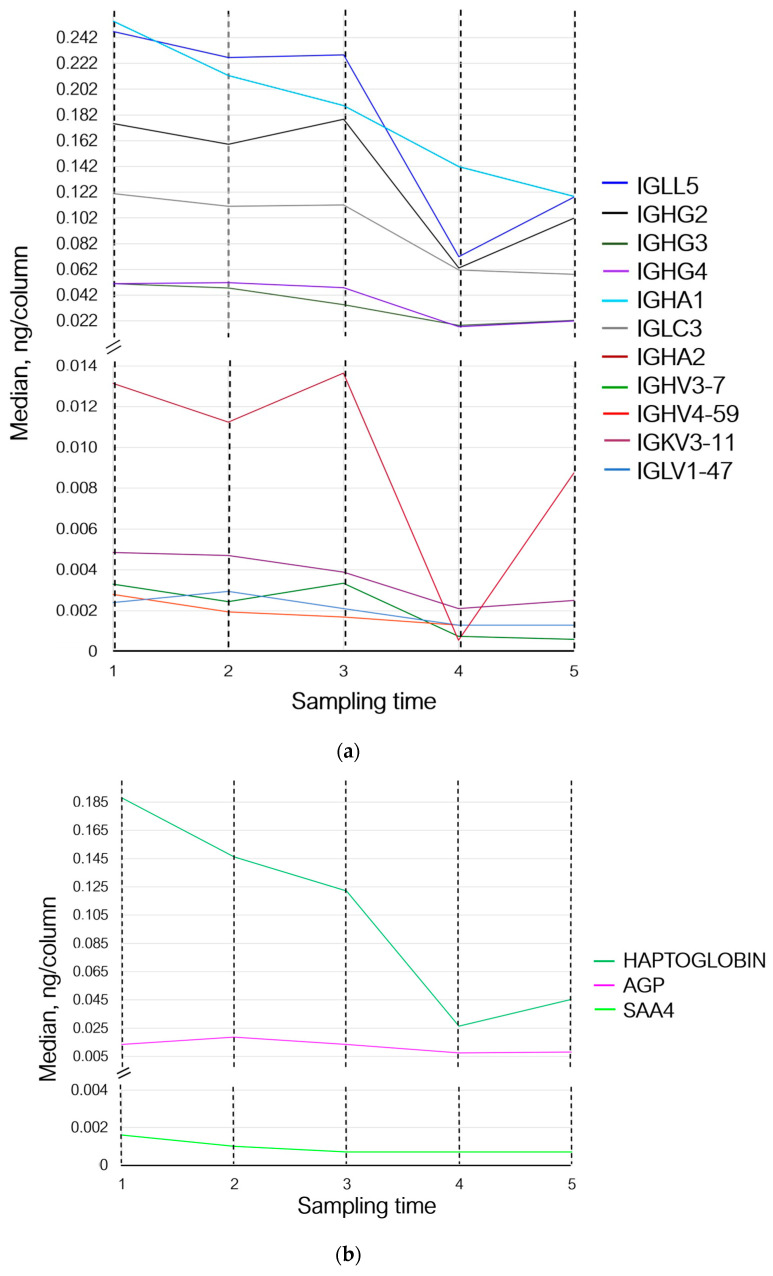

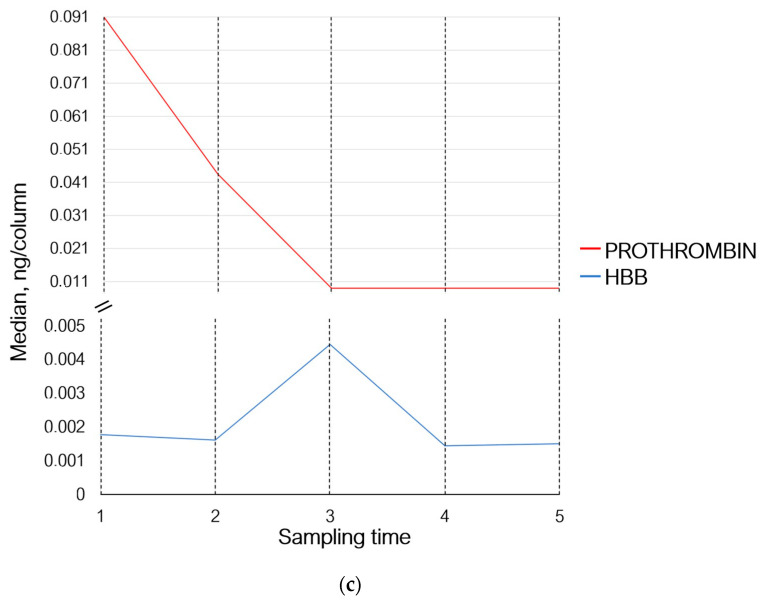
Variations in the levels of statistically significant proteins at sampling times 1–5, where (1) is the state before exercise was started, (2) is 30 min after exercise, (3) is 7 days after exercise, (4) is 14 days after exercise, and (5) is 21 days after exercise. (**a**) Changes in proteins associated with the adaptive immunity; (**b**) changes in inflammation-associated proteins; and (**c**) changes in proteins associated with homeostasis and oxygen transport. IGLL5—Ig lambda-like polypeptide 5; IGHG2—Ig heavy constant gamma 2; IGHG3—Ig heavy constant gamma 3; IGHG4—Ig heavy constant gamma 4; IGHA1—Ig heavy constant alpha 1; IGLC3—Ig lambda constant 3; IGHA2—Ig heavy constant alpha 2; IGHV3-7—Ig heavy variable 3–7; IGHV4-59—Ig heavy variable 4–59; IGKV3-11—Ig kappa variable 3–11; IGLV1-47—Ig lambda variable 1–47; AGP—alpha-1-acid glycoprotein 1; SAA4—serum amyloid A-4 protein; and HBB—hemoglobin subunit beta.

Thus, 30 min after the exercises (sampling time 2), the levels of proteins involved in the adaptive immunity either did not change or demonstrated multidirectional changes. The levels of proteins involved in homeostasis and inflammation (prothrombin, haptoglobin, hemoglobin subunit beta, and serum amyloid A-4 protein) decreased; the level of acute-phase protein alpha-1-acid glycoprotein 1, which participates in modulation of immune response, increased (Figure 3b,c).

Seven days (sampling time 3) after the exercises, concentrations of Ig lambda-like polypeptide 5 and Ig lambda constant 3 remained unchanged. The levels of proteins Ig heavy variable 3–7, Ig heavy constant gamma 2, and Ig heavy constant alpha 2 increased, while the level of Ig lambda variable 1–47 decreased and returned to the baseline (sampling time 1). The levels of Ig heavy variable 4–59, Ig heavy constant gamma 3, Ig heavy constant alpha 1, and Ig kappa variable 3–11 continued to decrease; the level of Ig heavy constant gamma 4 decreased slightly (Figure 3a). Concentrations of prothrombin, haptoglobin, and serum amyloid A-4 protein continued to decrease, while the level of alpha-1-acid glycoprotein 1 returned to the baseline values and the level of hemoglobin subunit beta increased (Figure 3b,c).

On day 14 of the recovery period (sampling time 4), the levels of Ig lambda-like polypeptide 5, Ig lambda variable 1–47, Ig heavy variable 4–59, Ig heavy constant gamma 2, and Ig heavy variable 3–7 decreased; the levels of Ig heavy constant gamma 3 and 4, Ig heavy constant alpha 1 and 2, Ig kappa variable 3–11, and Ig lambda constant 3 continued to decrease (Figure 3a). Concentrations of prothrombin and serum amyloid A-4 protein remained unchanged. The concentration of haptoglobin and alpha-1-acid glycoprotein 1 continued to decrease; the level of hemoglobin subunit beta returned to that observed on day 7 of the recovery period (sampling time 3) (Figure 3b,c).

By day 21 of the recovery period (sampling time 5), the levels of Ig heavy variable 3–7 and Ig lambda constant 3 had decreased slightly, and the concentration of Ig heavy constant alpha 1 continued to decline. Meanwhile, the levels of Ig lambda-like polypeptide 5, Ig heavy constant gamma 2, Ig heavy constant alpha 2, and Ig kappa variable 3–11 increased and the levels of Ig heavy constant gamma 3 and 4 tended to rise. The levels of Ig lambda variable 1–47 and Ig heavy variable 4–59 remained unchanged (Figure 3a).

The levels of prothrombin, serum amyloid A-4 protein, and alpha-1-acid glycoprotein 1 remained unchanged. The levels of hemoglobin subunit beta and haptoglobin increased (Figure 3b,c).

**Figure 4:**
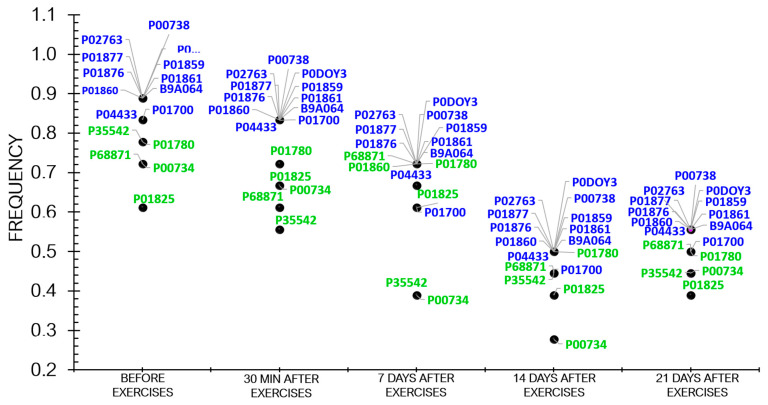
The dynamics of the frequency of statistically significant proteins in the athletes throughout the entire study.

Proteomic analysis revealed that the frequency of protein occurrence decreased as the athletes recovered (Figure 4).

Figure 4 demonstrates that the frequency of differentially expressed proteins (DEPs) tended to decline during the recovery period after physical load.

### 3.2. Metabolomic Analysis

**Figure 5:**
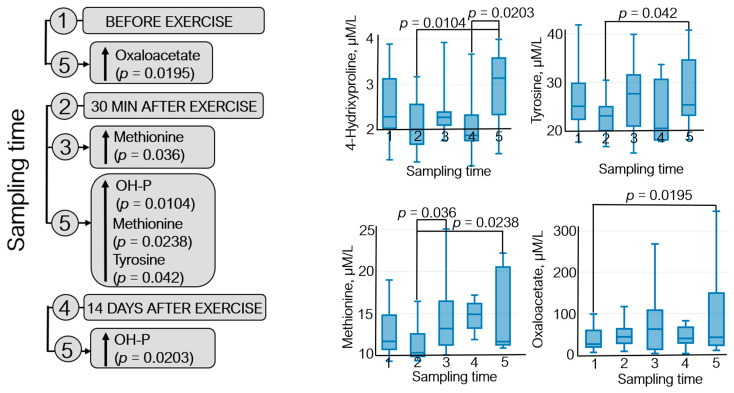
The dynamics of statistically significant variations in the levels of endogenous metabolites of the athletes throughout the entire study: (1) before exercise, (2) 30 min after exercise, (3) 7 days after exercise, (4) 14 days after exercise, and (5) 21 days after exercise. OH-P—4-hydroxyproline.

The metabolomic analysis revealed 22 metabolites (Figure S1); statistically significant variations in their levels were detected for four metabolites: 4-hydroxyproline, methionine, oxaloacetate, and tyrosine (Figure 5).

Figure 5 demonstrates that oxaloacetate level increased (~40%) during the recovery period (21 days after exercise) with respect to the pre-exercise level. An elevated methionine level was observed 7 days after exercise (↑~20%) and in the recovery period compared to that 30 min after exercise (↑~30%). Tyrosine level increased ~9% on day 21 after exercise with respect to that 30 min after exercise. Furthermore, the 4-hydroxyproline level was increased by 35–40% in athletes during the recovery period compared to that at sampling time 2 (30 min after exercise) and sampling time 4 (14 days after exercise).

### 3.3. Analysis of Blood Biochemistry Parameters

**Figure 6:**
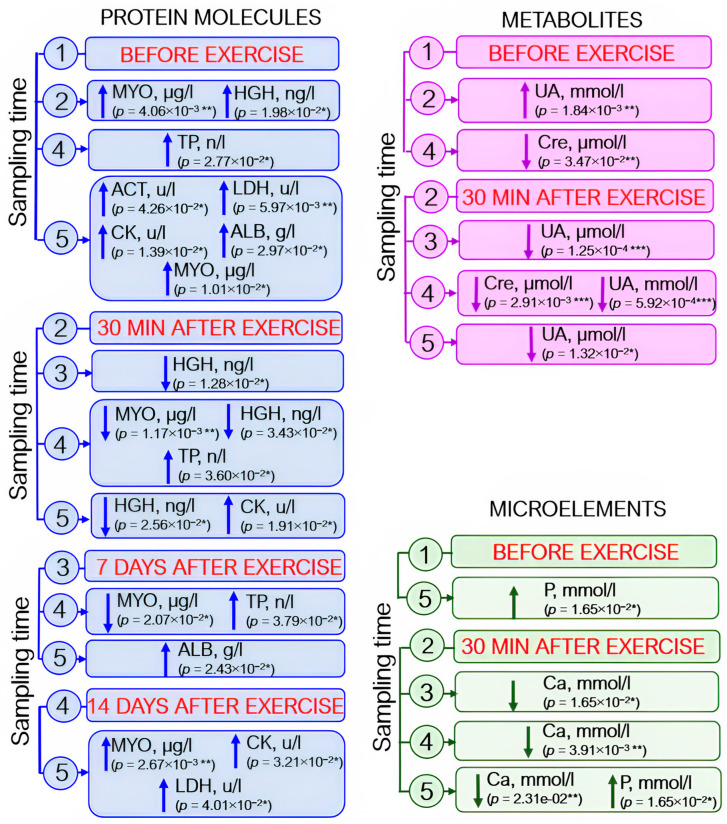
The dynamics of statistically significant variations in blood biochemistry parameters in the athletes throughout the entire study. ACT—aspartate aminotransferase; ALB—albumin; Ca—calcium; CK—creatine kinase; Cre—creatinine; HGH—human growth hormone; LDH—lactate dehydrogenase; MYO—myoglobin; P—phosphorus; TP—total protein; UA—uric acid. Sampling time: (1) athletes’ condition before exercise was started, (2) 30 min after exercise, (3) 7 days after exercise, (4) 14 days after exercise, and (5) 21 days after exercise. Symbol * – *p* < 0.05; ** – *p* < 0.01; *** – *p* < 0.001.

Analysis of blood biochemistry parameters revealed elevated enzyme levels: by 40–50% for creatine kinases and by 14% for aspartate aminotransferase; by day 21 after exercise, the level of lactate dehydrogenase had increased by 20% with respect to the pre-exercise level and by 12% with respect to that observed on day 14 after exercise. Albumin and total protein levels also rose (by ~5% and 1–3%, respectively). Myoglobin level increased by ~30% 30 min after load, decreased by day 14 of the recovery period (~50%), and further increased (~40%) by day 21 of the experiment. The somatotropin level rose almost fourfold 30 min after exercise and then returned to the baseline by day 7 (Figure 6, protein molecules).

Statistically significant changes in the levels of some metabolites were also identified. The creatinine level decreased by day 7 after exercise; on day 14 (the recovery period), creatinine level was reduced by ~7% with respect to the baseline level and the level recorded 30 min after exercise. By day 21 of the experiment, the baseline level tended to be restored, although this increase was not statistically significant. Uric acid concentration (↑~20%) increased as soon as after 30 min after exercise and then decreased by day 7 of the experiment. On day 21 of the recovery period, the uric acid level almost coincided with the baseline values (Figure 6, metabolites).

Among microelements, statistically significant variations in calcium and phosphorus levels were observed: increased calcium level was detected 30 min after exercise, but this change was not statistically significant (*p* = 0.075). Calcium concentration was significantly reduced on days 7, 14, and 21 after exercise with respect to its level 30 min after load (by 3%, 5%, and 3%, respectively). Phosphorus level rose gradually throughout the entire study and increased during the recovery period (on day 21 after load) by 14% with respect to the baseline (Figure 6, microelements).

### 3.4. Analysis of Complete Blood Count Parameters

The results of a complete blood count test were also analyzed in this study. The relative lymphocyte count 30 min after exercise (sampling time 2) was reduced (~35%) and subsequently increased by ~30% on days 7, 14, and 21 after exercise with respect to sampling time 2.

A similar pattern was observed for eosinophils and monocytes; the magnitude of variation was approximately 70% and 40%, respectively.

An opposite trend was found for white blood cells and neutrophils: Their levels increased 30 min after exercise (sampling time 2) and decreased on days 7, 14, and 21 after exercise with respect to sampling time 2.

Statistically significant variations in basophile level were observed on day 7 after exercise (sampling time 3) with respect to sampling time 2: The relative basophile count increased by ~40%.

The average concentration of hemoglobin in the erythrocyte increased 30 min after exercise; by day 21 of the recovery period, its level had decreased.

### 3.5. Individual Variations in Adaptation to Load under Hypoxic Conditions

Variations in individual response of the athletes’ bodies are observed for different training strategies, including altitude training; these variations are related to physiological and biochemical changes (red blood cell count, hemoglobin level, changes in cardiac output, the lactate threshold, heart rate response, etc.) and endurance [33].

Several factors associated with individual response to hypoxic training have been identified. However, research is still ongoing into the methods for identifying athletes who are most likely to benefit from hypoxic training. Physiological variations observed during hypoxic training are probably associated with genetic variations [34].

**Figure 7:**
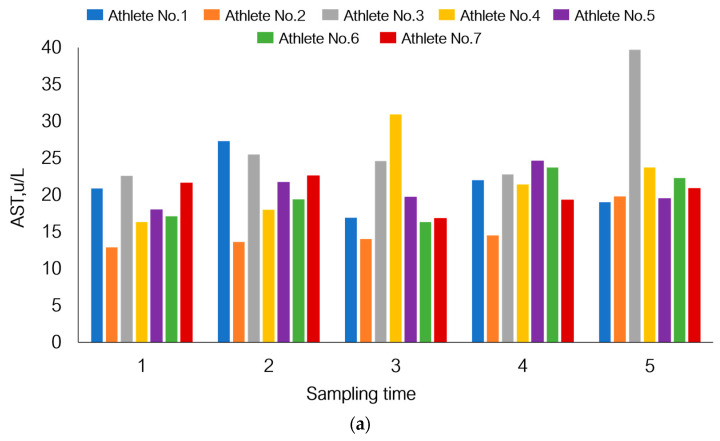

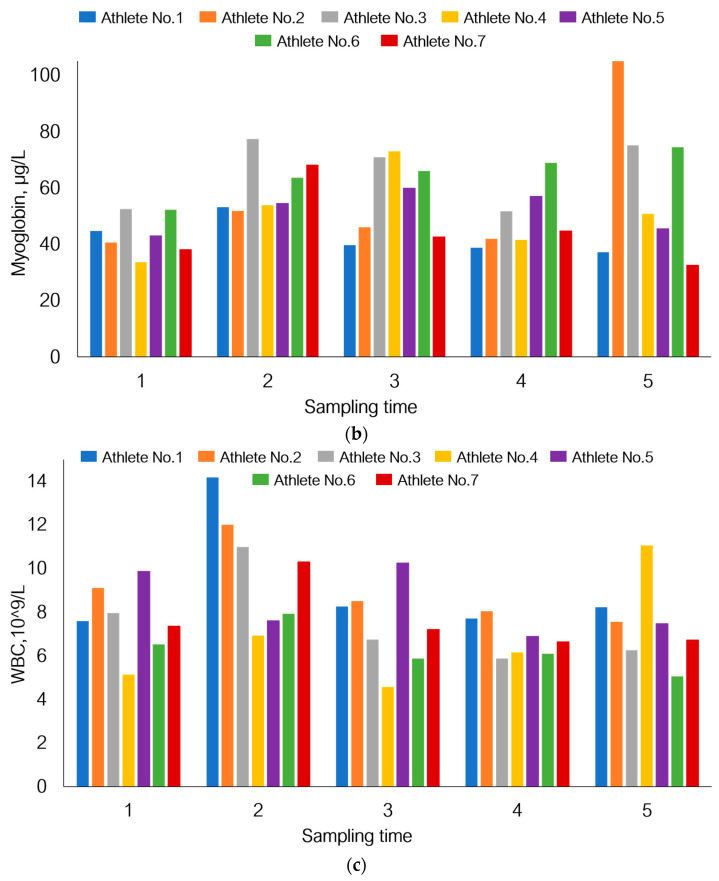
Individual response of the bodies of seven athletes participating in this study to physical load using changes in the levels of aspartate aminotransferase (**a**), myoglobin (**b**), and white blood cell count (**c**) under the given experimental conditions as an example.

If we focus on the response of athletes’ bodies to physical load at different sampling times in this study, the individual pattern of adaptive response will also be observed (Figure 7).

Although AST level in the total sample tended to increase by day 21, Figure 7 shows that athletes one and seven had reduced AST levels. A similar pattern was also observed for athletes one and seven in terms of myoglobin levels. The total white blood cell count declined by day 21 of the recovery period (*p* = 1.80 × 10^−3^), but on the contrary, this parameter was increased in athletes one and four.

Individual variations in the levels of analyzed biomolecules are rather diverse and are the personalized response to altitude and hypoxic training. Such changes can be associated with increased repression or transcription of an enormous number of genes [35]. The hypoxia-inducible factor 1 transcription factor is a pivotal element in these processes, and variations in the genes encoding hypoxia-inducible factor 1 [36] may affect athletes’ adaptation to altitude and performance. The genes involved in numerous responses to hypoxia, including those involved in recognizing changes in blood chemistry, respiratory impulse transduction, synthesis and degradation of muscle protein, cell substrate availability, cardiac contractility, and erythropoietic response, also play a crucial role.

## 4. Discussion

**Figure 8:**
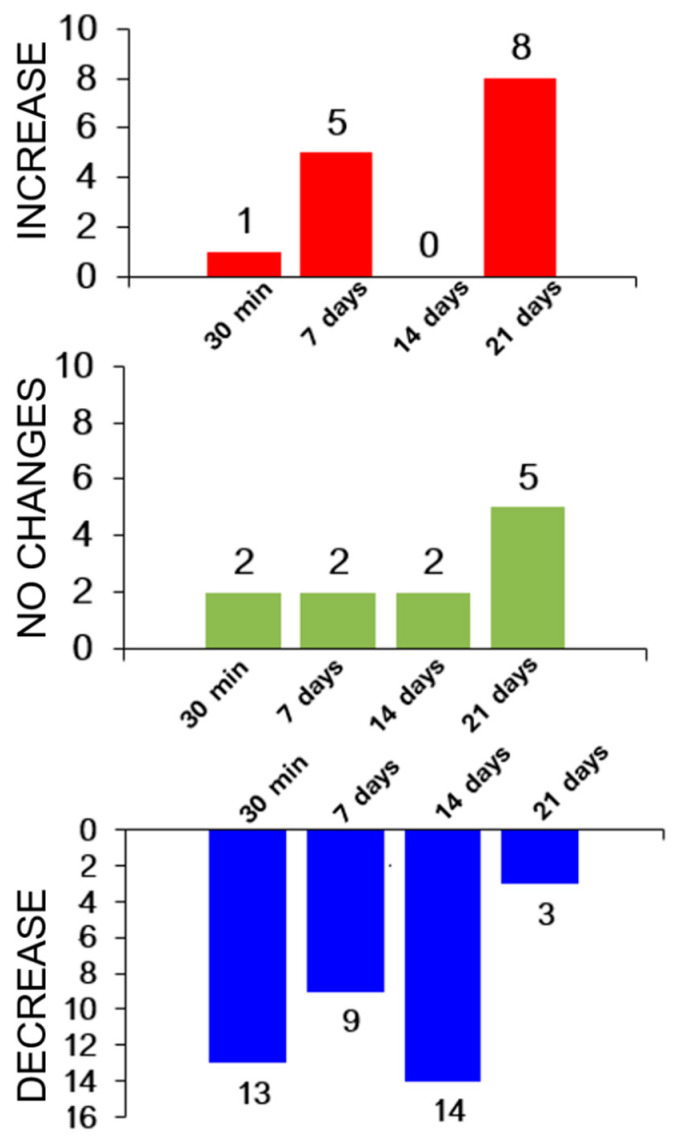
The general trend toward variation in protein levels in the experiment.

Proteomic analysis identified 16 proteins whose levels in the experiments differed statistically significantly from the baseline. In general, the levels of most proteins decreased throughout the entire study (Figure 8).

The baseline protein level tended to be restored only in the recovery period (21 days after exercise). This fact possibly indicates that recovery processes in an athlete’s body start to be intensified only on day 21 after exercise. However, elevated enzyme levels were still observed in the blood biochemistry data (Figure 6).

### 4.1. The Immune System and Exercise Load

Table 5 suggests that the biological role of most identified proteins consists of their involvement in adaptive immunity. It is known that circulating antibodies/immunoglobulins are usually associated with humoral adaptive immunity [37]. A significant discordance exists between the studies that focus on physical training and examine changes in immunoglobulin responses. Some studies have shown that immunoglobulin response increases [38,39,40] or decreases [41,42], while no variations have been revealed in other studies [43]. An interesting result was reported by McKune et al. [44], who showed that total serum IgG levels rise significantly immediately after exercise, accompanied by declining IgM and IgD levels.

It is impossible to identify the immunoglobulin class based on the results of our study, since immunoglobulins exist as different isoforms of light and heavy chains. However, the decrease in their levels after exercise and during the subsequent 14 days may be indirectly indicative of a reduction in immunoglobulin levels and, therefore, the development of immunosuppression [45].

**Figure 9:**
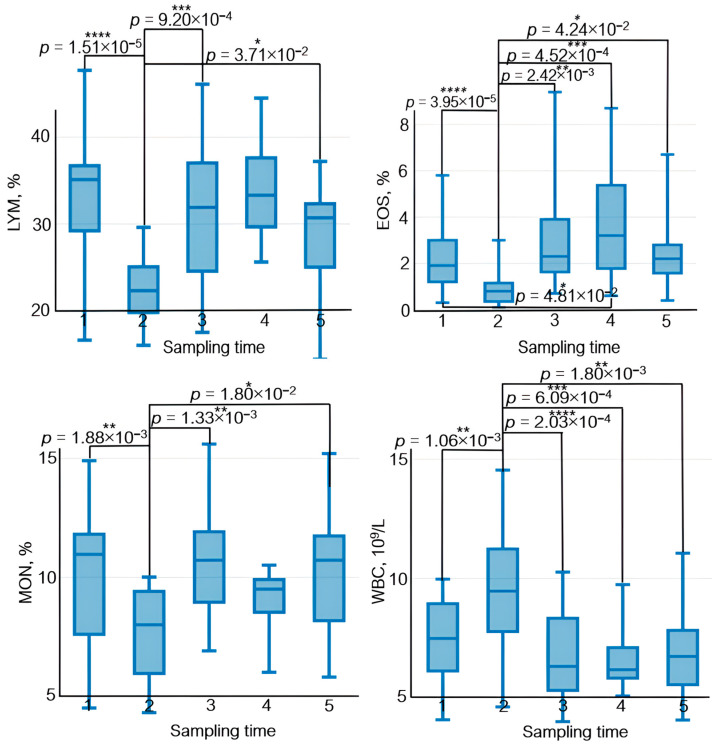

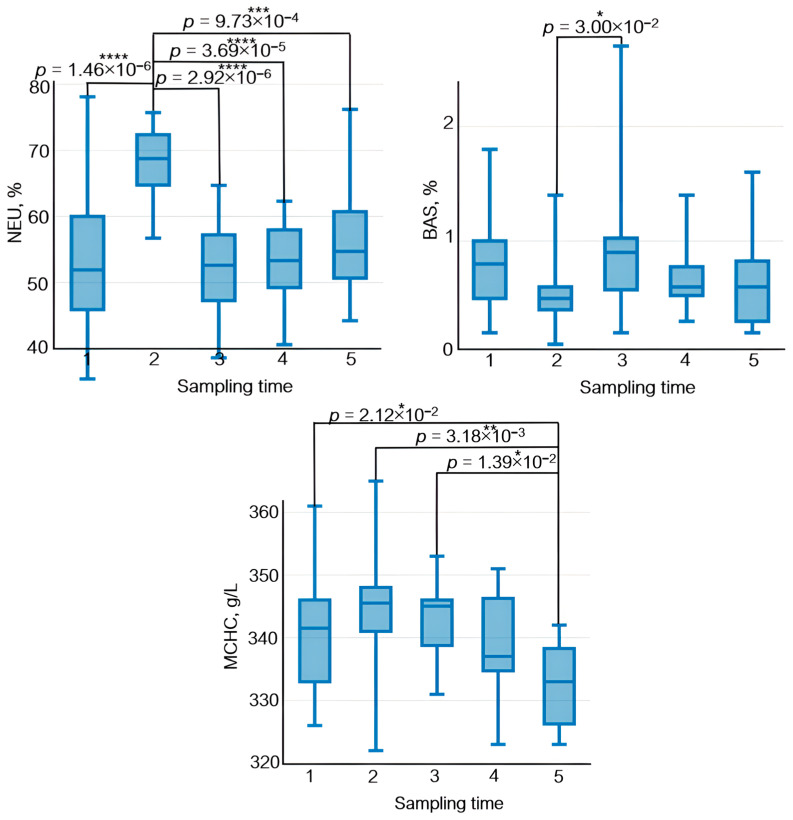
The dynamics of statistically significant variations in complete blood count parameters in the athletes throughout the entire study. BAS—basophils; EOS—eosinophils; LYM—lymphocytes; MCHC—the average concentration of hemoglobin in the erythrocyte; MON—monocytes; NEU—neutrophils; WBC—white blood cells. Sampling time: (1) athletes’ condition before exercise was started, (2) 30 min after exercise, (3) 7 days after exercise, (4) 14 days after exercise, and (5) 21 days after exercise. Symbol * – *p* < 0.05; ** – *p* < 0.01; *** – *p* < 0.001; **** – *p* < 0.0001.

The effect of physical load on the immune system manifests itself as variations in lymphocyte count, their subpopulation composition, and their functional activity. After physical load, lymphocyte count decreases rapidly; however, some publications suggest that this decrease is short term and that lymphocyte count in peripheral blood is normalized by the end of day 1 [46] (Figure 9).

Neutrophils, which are the essential components of natural immunity and ensure phagocytosis of bacteria and viruses as well as synthesis of immunoregulatory factors, are the first to respond to exercise among all the peripheral blood cells. Their count increases during exercise [47]. In our study, the neutrophil count also rose 30 min after physical load (Figure 9). Furthermore, physical training causes rapid monocyte recruitment in phagocytosis and upregulated expression of adhesion molecules [48].

### 4.2. Biomarkers for Oxygen Transport

Proteomic analysis also revealed that the hemoglobin level increased on day 7 after exercise and subsequently decreased. Similar variations in hemoglobin level were also demonstrated by blood biochemistry data (Table 3, Figure 3 and Figure 9). Measurements of hemoglobin levels have been used in sports for many years to monitor overtraining and performance in athletes [49,50]. The reasons for the reduced hemoglobin level can include impaired erythropoiesis because of fatigue and the slowing down of anabolism in the athlete’s body or iron deficiency caused by inadequate dietary iron intake and malabsorption of iron [50,51]. A reduced level of hemoglobin in the blood impairs the oxygen-carrying capacity and, therefore, worsens athletic performance, so monitoring its level is an efficient way of assessing sports performance.

### 4.3. Biomarkers for Muscle Damage and Inflammation

Muscle damage is rather common in many sports because of excessive stress during competitions or training. Muscle damage is accompanied by microtears, and the contents of some muscle fiber components get into the bloodstream. These cellular contents mainly consist of enzymes; if muscle damage is more severe, they may also contain some contractile proteins. Elevated levels of creatine kinase, aspartate aminotransferase, and lactate dehydrogenase were detected in our study 21 days after exercise (Figure 6), which may be indicative of muscle damage [50,52] and can be used for monitoring training load adequacy. A significant number of muscle microtraumas cause a greater level of creatine kinase secretion in the intercellular space [53]. Depending on the athlete’s individual characteristics, creatine kinase concentration changes during the first four days after a certain load [54,55] and is an indicator of the athlete’s training status and recovery [56]. In our study, elevated creatine kinase levels were observed on day 21 of the recovery period, thus probably indicating that a longer recovery period is needed. Thus, it was reported in [57,58,59] that it takes 24–120 h for the creatine kinase level to be normalized. In addition, the total creatine kinase activity depends on the individual’s age, sex, race, climatic conditions, training status, muscle groups involved in the exercise, and amount of strength training [54].

The significantly increased enzyme activity during the recovery period after training is an indicator of overtraining. In this case, coaches or sports medicine physicians should use proper recovery programs that involve dietary correction, physiotherapeutic techniques, specialized sports nutrition and food supplements, as well as pharmacotherapy. The international declaration on a four-factor strategy to optimize post-exercise recovery was published in 2021 [60]. If the existing recovery programs turn out to be inefficient, new recovery programs for athletes need to be elaborated. It is important that positive expert feedback be obtained for the recovery programs, which will indicate that it is reasonable and correct, as well as that it can be used during training. Meanwhile, one should bear in mind that decisions on training resumption should be made on a personalized basis, since there exist no absolute criteria for athletes’ complete recovery.

**Figure 10:**
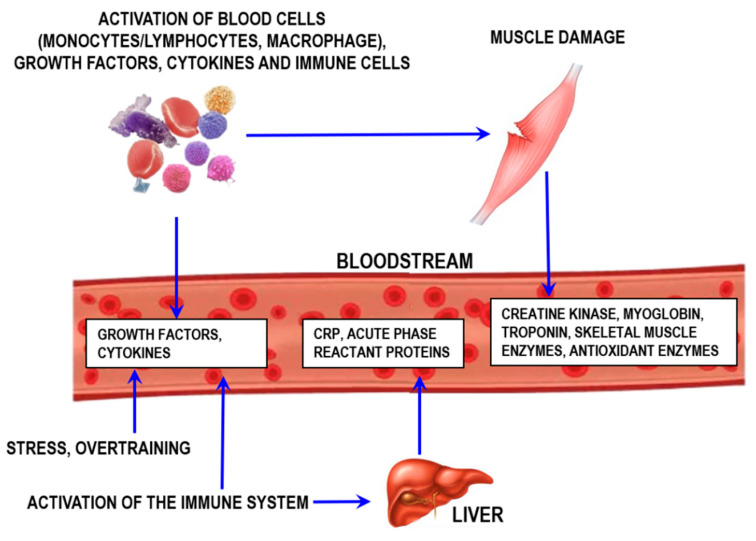
Schematic representation of muscle damage processes and exercise-induced inflammation. CRP—C-reactive protein.

Inflammation, which is indicative of injury or stress from either overtraining or infection/disease, can also be monitored by assessing proteins and other molecules controlling inflammation (Figure 10).

An important aspect to consider for the inflammatory process is that the local response is usually followed by the systemic inflammatory response (the acute phase) [61]. One of the key acute-phase responses involves increasing synthesis of proteins [62]. The most prominent of these proteins include C-reactive protein, α-1-acid glycoprotein, serum amyloid A, haptoglobin, fibrinogen, transferrin, and ceruloplasmin [62,63]. Each of them exerts specific functions in the inflammatory process.

Proteomic analysis revealed that the levels of serum amyloid A decreased 30 min and 7 days after exercise while subsequently remaining unchanged (Figure 3, Table 5). This discrepancy can be related to the smaller sample size in the present study, although some publications show that the levels of acute-phase proteins decrease as infection or injury is resolved [64]. Still, larger-scale studies assessing changes in serum amyloid A levels after exercise in highly trained athletes need to be performed.

Haptoglobin is a highly conserved acute-phase protein that responds to infection and inflammation [65]. The most prominent biological functions of haptoglobin are scavenging of released hemoglobin and involvement in oxidative stress [66]. In this study, like for most proteins, we observed a reduction in haptoglobin level after exercise and a trend towards recovery 21 days after the physical load (Figure 3b). The changes in haptoglobin levels can be caused by the fact that exercise induces a chronic hemolytic reaction [67]. Nevertheless, there still are ongoing controversial debates about the effect of exercise on haptoglobin expression; however, it is noteworthy that in general, the increase in haptoglobin expression in response to exercise occurs rather slowly and is observed 5 days after a single exercise session [68].

On the other hand, there are some proteins whose levels decrease in the acute phase; albumin is among such proteins [63]. A blood biochemistry test showed that albumin levels increased statistically significantly during the recovery period (21 days after exercise) (Figure 6). Studies performed by other research groups demonstrated that plasma albumin level rose within 1 h after strenuous activity; however, albumin synthesis decreased after 1–5 h of recovery and then increased only after 21–22 h of recovery [69].

Myoglobin release is a short-term marker of damage that can be measured in blood [70]. Although myoglobin levels increased 30 min after exercise, they remained elevated during the recovery period (21 days after exercise) in our study (Figure 6).

Activation of inflammation and muscle tissue damage during exercise that often accompany physical activity are also associated with cytokine production [71]. Blood supply to muscle capillaries and exercise-induced angiogenesis are partially regulated by vascular endothelial growth factor (VEGF). VEGF is produced by skeletal muscle cells and can be secreted straight into the bloodstream [72].

Erythropoietin (EPO) is known to improve physical performance by enhancing blood oxygen transport and thus inducing higher maximal oxygen uptake. However, in our study, enzyme-linked immunoassay of athletes’ plasma to detect elevated levels of IFNα, IL18, TNFα, VEGF, or EPO revealed no statistically significant results (Supplementary Materials).

### 4.4. Biomarkers for Metabolic Fitness and Performance

The demand for ATP synthesis increases during strenuous exercise, and glucose becomes the predominant muscle substrate for it. Since glycolysis leading to pyruvate formation is intensified, lactate production is also enhanced. Blood biochemistry data showed that the lactate dehydrogenase level increased 21 days after exercise. This can be an indirect sign of lactate formation, since the reduction of pyruvate to lactate is catalyzed by lactate dehydrogenase, thus increasing the cytosolic lactate concentration.

Lactate buildup is associated with excessive accumulation of H^+^ ions, which in turn compete with calcium (Ca^2+^) for the troponin C binding site, thus impeding muscle contraction [73,74]. In our study, the Ca^2+^ level decreased 7 days after exercise with respect to that 30 min after exercise (Figure 6).

In this study, phosphorus content after the exercise increased (Figure 6), which is consistent with findings reported in other publications [75,76]. The increased phosphorus level is possibly associated with the fact that phosphorus compounds in muscles are components of the adenylate energy system (AMP, ADP, ATP, and creatine phosphate) and participate in energy metabolism during muscle activity.

Strenuous exercise aggravates oxidative stress; reactive oxygen species are produced, and free radicals are vigorously released. Methionine is believed to exhibit antioxidant properties, partly due to the sulfur atom [77], and an increase in methionine level (Figure 5) is potentially indicative of changes in oxidative stress and signaling pathways in athletes [78].

Changes in concentrations of intermediates of the TCA cycle (e.g., oxaloacetate) (Figure 5) probably occur because higher energy demand needs to be met during exercise [79].

### 4.5. Hemostasis and Physical Training

Hemostasis is achieved due to the delicate balance between coagulation and fibrinolytic cascades. All the components of these cascades exist in the bloodstream as inactive proteins, which are converted into the active enzymatic form (prothrombin → thrombin → fibrinogen → fibrin) once the cascades are activated. Prothrombin time is an indicator of the blood level of prothrombin. 

Along with changes in the overall coagulation activity and faster prothrombin activation during training episodes, the factor VIII level and activity of factor XII (a trigger of the intrinsic prothrombin activation pathway) can also increase. Simultaneously, antithrombin activity somewhat increases, while heparin level and fibrinolysis decrease. Meanwhile, adaptation to repeated physical activity eventually leads to marked hypocoagulation, with accumulation of fibrinogen and fibrin degradation products being one of the reasons for that. Factors V, VII, and VIII, which are directly involved in prothrombin conversion, play a crucial role in elevation of hypercoagulemic activity during individual episodes of high-intensity muscle work, along with activation of factor XII. In most cases, the detected hypercoagulation state is physiologically normal. However, fibrinolytic activity of blood is enhanced during muscular work, together with increasing coagulation ability of plasma, mainly due to active plasminogen release. It has been found that after 10–15 min of a single training session on a stationary bicycle, fibrinopeptide A level does not increase despite the activation of coagulation and fibrinolysis. Platelet aggregation ability can also increase during an appreciably strenuous single training session [79,80]. Nonspecific changes, including those in the blood aggregate state regulation system, arise from exposure to any high-intensity factor and are accompanied by rearrangement of the defense system. During the alarm-stage response, at the stage of anxiety, the defense mechanisms are mobilized and transfer the body to the resistance stage. Hypoxia is an important reason for developing hypercoagulability during high-intensity muscle activity; its level increases with the output of work performed. Hypoxia is accompanied by an increased release of catecholamine into the blood, and adrenaline in particular (elevation in its concentration is accompanied by coagulation activation). Catecholamines also play a crucial role in increasing the platelet aggregation ability [81].

In our study, we observed a gradual reduction in prothrombin level 30 min and 7 days after exercise; this level was subsequently maintained until the end of the experiment (Figure 3, Table 5). The results of other studies are rather ambiguous and show either that prothrombin time in athletes increases after running [82] or that plasma levels of fibrinogen, plasminogen, or prothrombin remain unchanged [83].

In some cases, regular physical activity may affect the lipid composition of platelet membranes, which consists of a reduction in cholesterol loading of platelet membranes and a decrease in the gradient of cholesterol/total phospholipids in platelets [81]. There is evidence that controlled physical activity positively affects the platelet activity that was increased at baseline. Training was found to normalize platelet adhesion, thus being indicative of restoration of elasticity of microvessels and prevention of their contact with collagen fibers of the blood vessel wall [84].

Physical activity stimulates both coagulation and fibrinolysis [85]. There is a common opinion that exercise is beneficial for health and favorably affects wellbeing, including the blood lipid profile, cardiovascular diseases, etc. However, the question regarding the optimal amount and intensity of physical activity for an individual still remains relevant. Very strenuous and/or intense physical activity such as marathon running can be ineffective for some individuals; heterogeneous activation of the coagulation and fibrinolytic cascades can be one of the mechanisms for that.

### 4.6. Health Risks for Athletes Performing Physical Activity under Hypoxic Conditions

The altitude where athletes usually compete or train typically is no higher than 2000–2500 m. At these moderate altitudes, acute mountain sickness occurs in mild form. Furthermore, people develop neither cerebral nor pulmonary edema at these altitudes, and periodic exposure to much higher altitudes (4000–6000 m) used by athletes for hypoxic training is too short to cause acute pathological processes [86].

It is also worth mentioning that pronounced immunological changes observed during hypoxic training may partly result from increased work intensity. Furthermore, hypoxemia directly regulates various immune functions (macrophage migration and phagocytosis) [87]. Hypoxia-inducible factor 1 can be activated by physical activity [88], thus enhancing immune responses to training under hypoxic conditions. A set of molecular scenarios, physical training, and hypoxia, which have direct consequences for immune function and recovery, occur at the level of mitochondrial adaptation and oxidative stress/antioxidant mechanisms. It is known that excessive training may cause mitochondrial dysfunction [89].

However, even moderate altitudes between 2000 and 3000 m can cause exacerbation of cardiovascular or pulmonary diseases or induce the onset of a previously undiagnosed disease, especially in elderly people who may belong to athlete support personnel. Moderate altitudes can also cause spleen infarction in healthy athletes with sickle cell anemia [86].

Therefore, coordination of and a gradual increase in training intensity and hypoxia level, together with regular monitoring of individual physiological responses, are critical for optimal exercise performance under hypoxic conditions and athlete health.

### 4.7. The Prospects for the Research into Novel Biomarkers

Proteins are considered key biomarkers for monitoring training process. Due to their multiple functional roles as enzymes, cell signaling molecules, cofactors, and neurotransmitters, many of these factors are indicative of stress and recovery, as well as adaptation processes. High-throughput technologies using multiplex assays or mass spectrometry can be used to identify potential candidate proteins [70,90].

Metabolomics allows one to assess the variations indicating different physiological and pathophysiological states of athletes, suggesting that identification of metabolic signatures can be used to monitor athletes’ health, response to training, and performance [91]. However, when analyzing the metabolome, it is important to take into account such factors as diet, training regime and type, as well as the circadian rhythm.

Some companies currently offer commercial products for metabolomic and proteomic analysis of athletes’ bodies (Table 6).

Currently, there are several approaches to organizing training under hypoxic conditions: (1) Athletes live and train at moderate altitudes (2000–2500 m) for up to 6 weeks. This adaptation usually lasts for 1–2 weeks after returning to sea level; the athlete participates in several major competitions during this period. (2) Athletes live at sea level but train at altitude. (3) Athletes live at altitude for several weeks but then return to sea level for most of their training [33]. There is also a training program developed by the Australian Institute of Sport that requires athletes to be exposed to altitude/hypoxia by living in a simulated altitude environment (14 h per day) or being exposed to intermittent hypoxia [92]. However, the efficiency and performance of training at moderate and low altitudes is dubious and requires further investigation [93].

The integration of omics approaches, including genomics, metabolomics, and proteomics (sportomics), can be used to understand the metabolism during training under the real-world conditions experienced by athletes, which would allow one to potentially conduct personalized interventions to improve athletes’ performance and recovery as well as reduce injury rate.

In the long run, multiomic studies in combination with machine learning and bioinformatics approaches will allow one to develop recommendations and measures to improve acclimatization, tolerance, and safety of hypoxic training, taking into account athletes’ individual molecular profiles.

## 5. Conclusions

Research into biomarkers (their identification and interpretation) for monitoring the performance, overtraining, and general wellbeing of athletes has been conducted for several decades, but the results are insufficient to be used in practice. Proteomics and the relatively new field, metabolomics, offer tremendous opportunities to improve the understanding of performance and recovery processes in athletes as well as their adaptation to physical training.

In the current study, changes in the athletes’ bodies occurring during 21 days after exercise were revealed by proteomic and metabolomic analyses as well as a blood chemistry test. The identified statistically significant changes were mainly related to tissue damage, activation of the immune system and inflammation, homeostasis, and metabolic activity of the body.

Assessing changes in biomarker levels after exercise is critical for identifying the potential source of the physiological/pathological process. This will enable further actions to be identified to maintain athletes’ health and achieve optimal performance.

## 6. Limitations

In this study, athletes did not fill out questionnaires that would allow us to find out whether they used any recovery strategies (such as massage, specialized nutrition, taping, etc.) after the exercise.

## Figures and Tables

**Table 1:** Molecular research methods that can be used at different stages of athlete selection and orientation.

Stages	Stage Objectives [28]	Methods
Primary stage	Elucidating whether mastering in a particular sport is reasonable.	Genomic methods (analysis of the markers of sports aptitude and developing occupational diseases).
Preliminary stage	Finding out whether a person has the potential to efficiently master a sport. Choosing a sports specialization.	Genomic (determining the potential of developing physical qualities, identifying the strengths and weaknesses of one’s body) and epigenomic methods.
Intermediate stage	Finding out whether a person has the potential of showing high sports performance and enduring strenuous training and competition load.	Pharmacogenomic, nutrigenomic, metagenomic, and standard biochemical methods; analysis of circulating DNA.
Main stage	Finding out whether a person has the potential to achieve international-level results. Elaborating the strategy and approaches to training and competitive activity.	Transcriptomic, proteomic, metabolomic, and standard biochemical methods.
Final stage	Finding out whether a person can maintain and improve the results achieved. Elucidating on whether it is reasonable to continue the athletic career.	Measuring telomere length and telomerase activity; transcriptomic, proteomic, metabolomic, and standard biochemical methods.

**Table 2:** Anthropometric characteristics of the study participants.

Sample No. #	Age (years)	Weight (kg)	Height (cm)	BMI (kg/m^2^)
1	37	81	176	26.1
2	31	74	172	25
3	24	69	175	22.5
4	30	87	175	28.4
5	28	76	179	23.8
6	24	80	178	25.2
7	30	74	177	23.6
8	31	73	172	24.7
9	33	91	187	26
10	37	86	186	24.9
11	30	99	193	26.6
12	33	95	182	28.7
13	31	81	176	26.1
14	29	87	187	24.9
15	40	74	172	25
16	35	81	176	26.1
17	25	89	190	24.7
18	24	61	174	20.1

#: The sample number.

**Table 3:** Functional indicators of the study participants.

Indicators	Units	Mean (SD)
VO_2_ max	mL/min/kg	51 [47.5–54.4]
RR	amp	54.9 [48.4–61.4]
Resting heart rate	bpm	190 [185–195]
RER	relative units	1.11 [1.09–1.14]
Aerobic threshold		
VO_2_	mL/min	29.5 [26.6–32.5]
VE	L/min	60.3 [51.5–69.1]
Heart rate	bpm	129.2 [119–138]
Aanaerobic threshold		
VO_2_	mL/min	48.1 [45.2–50.9]
VE	L/min	125.6 [117.4–133.8]
Heart rate	bpm	179 [174–184]

Abbreviations: amp—actions per minute; bpm—beats per minute; RER—respiratory exchange ratio, RR—respiratory rate; RQ—respiratory quotient; VE—respiratory minute volume; VO_2—_oxygen consumption.

**Table 4:** Biochemical parameters of venous blood and their reference values.

Parameter	Reference Values	Unit of Measure
Total protein	66–87	g/L
Albumin	39.7–49.4	g/L
Creatinine	62–106	μmol/L
Urea	2.76–8.07	mmol/L
Uric acid	202–416	mmol/L
Triglycerides	0.4–1.7	mmol/L
Total cholesterol	3.9–5.2	mmol/L
High-density lipoprotein cholesterol	0.9–1.45	mmol/L
Low-density lipoprotein cholesterol	0.26–2.6	mmol/L
Total bilirubin	5–21	μmol/L
Direct bilirubin	0–5.1	μmol/L
Alanine aminotransferase (ALT)	5–41	U/L
Aspartate aminotransferase (AST)	5–40	U/L
Creatine kinase (CK)	7–190	U/L
Creatine kinase—MB (CK-MB)	3–25	U/L
Lactate dehydrogenase (LDH)	135–225	U/L
Gamma GT (GGT)	10–60	U/L
Alkaline phosphatase (ALP)	35–130	U/L
Lactate	0.5–2.2	mmol/L
Amylase	28–100	U/L
Total calcium	2.1–2.6	mmol/L
Phosphorus	0.81–1.45	mmol/L
Magnesium	0.66–1.07	mmol/L
Iron	5.83–34.5	μmol/L
Acid phosphatase	0.5–6.6	U/L
Somatotropic hormone (STH)	0.03–2.47	ng/mL
Total testosterone	8.64–29	nmol/L
Cortisol	171–536	nmol/L
Thyrotropic hormone (TSH)	0.27–4.2	μME/mL
Free thyroxine (free T4)	12–22	pmol/L
Myoglobin	23–72	µg/L

**Table 5:** Differentially expressed proteins significantly differing between the comparison groups in terms of their level before physical activity was started (|FC| > 2, *p* < 0.05).

UniProt ID	Protein Name	Sampling Time 2		Sampling Time 3		Sampling Time 4		Sampling Time 5		Biological Process
		FC	Log FC	FC	Log FC	FC	Log FC	FC	Log FC	
B9A064	Ig lambda-like polypeptide 5	0.91	−0.12	0.92	−0.11	0.28	−1.78	0.47	−1.06	Adaptive immunity
P00734	Prothrombin	0.75	−0.41	0.47	−1.08	0.47	−1.08	0.47	−1.08	Homeostasis
P00738	Haptoglobin	0.77	−0.36	0.64	−0.62	0.14	−2.83	0.24	−2.05	An acute inflammatory response
P01700	Ig lambda variable 1–47	1.22	0.29	0.87	−0.19	0.54	−0.88	0.54	−0.88	Adaptive immunity
P01780	Ig heavy variable 3–7	0.74	−0.42	1.01	0.02	0.22	−2.13	0.18	−2.45	Adaptive immunity
P01825	Ig heavy variable 4–59	0.69	−0.52	0.60	−0.71	0.46	−1.10	0.46	−1.10	Adaptive immunity
P01859	Ig heavy constant gamma 2	0.90	−0.14	1.01	0.02	0.35	−1.48	0.57	−0.78	Adaptive immunity
P01860	Ig heavy constant gamma 3	0.93	−0.10	0.67	−0.56	0.35	−1.48	0.43	−1.20	Adaptive immunity
P01861	Ig heavy constant gamma 4	1.01	0.02	0.93	−0.09	0.33	−1.56	0.42	−1.24	Adaptive immunity
P01876	Ig heavy constant alpha 1	0.83	−0.26	0.74	−0.43	0.55	−0.84	0.46	−1.10	Adaptive immunity
P01877	Ig heavy constant alpha 2	0.85	−0.22	1.03	0.05	0.41	−1.25	0.66	−0.58	Adaptive immunity
P02763	Alpha-1-acid glycoprotein 1	1.37	0.46	0.54	−0.86	0.60	−0.72	0.99	−0.00	An acute inflammatory response
P04433	Ig kappa variable 3–11	0.96	−0.45	0.80	−0.31	0.43	−1.20	0.51	−0.95	Adaptive immunity
P0DOY3	Ig lambda constant 3	0.91	−0.12	0.92	−0.11	0.50	−0.97	0.47	−1.06	Adaptive immunity
P35542	Serum amyloid A-4 protein	0.62	−0.67	0.43	−1.19	0.43	−1.19	0.43	−0.19	An acute inflammatory response
P68871	Hemoglobin subunit beta	0.90	−0.14	2.5	1.32	0.81	−0.29	0.84	−0.24	Oxygen transport

**Table 6:** Selected companies offering services for protein and metabolite identification in athletes.

Company	Parameters	Identification Method	Biomaterial Type	Website
INVITRO (Moscow, Russia)	Plasma amino acids (48 parameters)	HPLC, MS/MS	Plasma (EDTA)	https://www.invitro.ru/en/about/ (accessed on 20 February 2024)
Chromsystems (Gräfelfing/ Munich, Germany)	Organic acids (60 parameters); amino acids (48 parameters); vitamins B3, B6, B9, B12, D; hormones and metabolites (18 parameters); acylcarnitines (15 parameters); vitamins A, E, C; coenzyme Q10	HPLC-MS, LFIA, GC-MS, HPLC-FC, RPC	Blood (EDTA) Urine Plasma (EDTA) Serum	https://chromsystems.com/ (accessed on 20 February 2024)
Helix (Moscow, Russia)	Magnesium, transferrin, calcium, potassium, sodium, chlorine, zinc, vitamin B6, vitamin D, vitamin E. Cortisol, testosterone, LH, progesterone, T3, T4, TSH, FSH, estradiol, prolactin	Colorimetric analysis, immunoturbidimetry, ion-selective electrodes, HPLC, ECLIA, HPLC-MS	Venous blood	https://helix.ru/ (accessed on 20 February 2024)
AxisPharm (San Diego, CA, USA)	Amino acids, carbohydrates, alcohols, organic acids, amines, Krebs cycle intermediates, lipid macromolecules	GC-MS, LC-MS, NMR	Biofluids, tissues, cell cultures	https://axispharm.com/ (accessed on 20 February 2024)
MS-Omics (Vedbæk, Denmark)	Short-chain fatty acids, volatile polar and semi-polar metabolites, amino acids and organic acids, bile acids, lipids	GC-MS/MS, GC-MS	–	https://www.msomics.com/ (accessed on 20 February 2024)
EMBL (Hamburg, Germany)	Non-targeted metabolomics, targeted metabolomics, lipidomics, analysis of drugs and their metabolites	LC-MS/MS, HRMS in the mode of positive and/or negative ionization coupled with UHPLC in the HILIC mode, HRMS coupled with UHPLC in the RPC mode.	Cell cultures, tissues, plasma, serum, urine	https://www.embl.org/ (accessed on 20 February 2024)

ECLIA—electrochemiluminescence immunoassay; GC–MS—gas chromatography–mass spectrometry; GC-MS/MS—gas chromatography–tandem mass spectrometry; HPLC—high-performance liquid chromatography; HPLC-FC—high-performance liquid chromatography with fluorescent detection; HPLC-MS—high-performance liquid chromatography coupled with mass spectrometry; HRMS—high-resolution mass spectrometry; LH—luteinizing hormone; LFIA—lateral flow immunoassay; LC–MS—liquid chromatography–mass spectrometry; LC-MS/MS—liquid chromatography–tandem mass spectrometry; MS/MS—tandem mass spectrometry; NMR—nuclear magnetic resonance; UHPLC—ultra-high-performance liquid chromatography; RPC—reversed-phase chromatography; T3—free triiodothyronine; T4—free thyroxine; TSH—thyroid-stimulating hormone; FSH—follicle-stimulating hormone.

## Data Availability

The datasets generated and/or analyzed during the current study are available from Kristina A. Malsagova (kristina.malsagova86@gmail.com) on reasonable request.

## Supplementary Materials

The following supporting information can be downloaded at: https://www.mdpi.com/article/10.3390/sports12030072/s1, Figure S1: Results of quantitative determination of the content of erythropoietin (a), alpha-interferon (b), interleikin-18 (c), tumor necrosis factor (d), and vascular endothelial growth factor (e) in the blood plasma of the athletes.

## References

[1] Malsagova K.A., Butkova T.V., Kopylov A.T., Izotov A.A., Rudnev V.R., Klyuchnikov M.S., Stepanov A.A., Kaysheva A.L. (2021). Molecular Portrait of an Athlete. Diagnostics.

[2] Contrepois K., Wu S., Moneghetti K.J., Hornburg D., Ahadi S., Tsai M.-S., Metwally A.A., Wei E., Lee-McMullen B., Quijada J.V. (2020). Molecular Choreography of Acute Exercise. Cell.

[3] Haller N., Reichel T., Zimmer P., Behringer M., Wahl P., Stöggl T., Krüger K., Simon P. (2023). Blood-Based Biomarkers for Managing Workload in Athletes: Perspectives for Research on Emerging Biomarkers. Sports Med..

[4] Elrayess M.A., Botrè F., Palermo A. (2022). Editorial: OMICS-Based Approaches in Sports Research. Front. Mol. Biosci..

[5] Pitsiladis Y.P., Tanaka M., Eynon N., Bouchard C., North K.N., Williams A.G., Collins M., Moran C.N., Britton S.L., Fuku N. (2016). Athlome Project Consortium: A Concerted Effort to Discover Genomic and Other “Omic” Markers of Athletic Performance. Physiol. Genomics.

[6] Tanisawa K., Wang G., Seto J., Verdouka I., Twycross-Lewis R., Karanikolou A., Tanaka M., Borjesson M., Di Luigi L., Dohi M. (2020). Sport and Exercise Genomics: The FIMS 2019 Consensus Statement Update. Br. J. Sports Med..

[7] Sellami M., Bragazzi N.L. (2021). The Effect of Sport and Physical Activity on Transport Proteins: Implications for Cancer Prevention and Control. Adv. Protein Chem. Struct. Biol..

[8] Williams S.A., Kivimaki M., Langenberg C., Hingorani A.D., Casas J.P., Bouchard C., Jonasson C., Sarzynski M.A., Shipley M.J., Alexander L. (2019). Plasma Protein Patterns as Comprehensive Indicators of Health. Nat. Med..

[9] Chow L.S., Gerszten R.E., Taylor J.M., Pedersen B.K., van Praag H., Trappe S., Febbraio M.A., Galis Z.S., Gao Y., Haus J.M. (2022). Exerkines in Health, Resilience and Disease. Nat. Rev. Endocrinol..

[10] Robbins J.M., Rao P., Deng S., Keyes M.J., Tahir U.A., Katz D.H., Beltran P.M.J., Marchildon F., Barber J.L., Peterson B. (2023). Plasma Proteomic Changes in Response to Exercise Training Are Associated with Cardiorespiratory Fitness Adaptations. JCI Insight.

[11] Guseh J.S., Churchill T.W., Yeri A., Lo C., Brown M., Houstis N.E., Aragam K.G., Lieberman D.E., Rosenzweig A., Baggish A.L. (2020). An Expanded Repertoire of Intensity-Dependent Exercise-Responsive Plasma Proteins Tied to Loci of Human Disease Risk. Sci. Rep..

[12] Kurgan N., Noaman N., Pergande M.R., Cologna S.M., Coorssen J.R., Klentrou P. (2019). Changes to the Human Serum Proteome in Response to High Intensity Interval Exercise: A Sequential Top-Down Proteomic Analysis. Front. Physiol..

[13] Mi M.Y., Barber J.L., Rao P., Farrell L.A., Sarzynski M.A., Bouchard C., Robbins J.M., Gerszten R.E. (2023). Plasma Proteomic Kinetics in Response to Acute Exercise. Mol. Cell. Proteomics.

[14] Poortmans J., Jeanloz R.W. (1968). Quantitative Immunological Determination of 12 Plasma Proteins Excreted in Human Urine Collected before and after Exercise. J. Clin. Investig..

[15] Kristensen J.H., Karsdal M.A., Genovese F., Johnson S., Svensson B., Jacobsen S., Hägglund P., Leeming D.J. (2014). The Role of Extracellular Matrix Quality in Pulmonary Fibrosis. Respir. Int. Rev. Thorac. Dis..

[16] Genovese F., Karsdal M.A. (2016). Protein Degradation Fragments as Diagnostic and Prognostic Biomarkers of Connective Tissue Diseases: Understanding the Extracellular Matrix Message and Implication for Current and Future Serological Biomarkers. Expert Rev. Proteomics.

[17] Bongiovanni T., Pintus R., Dessì A., Noto A., Sardo S., Finco G., Corsello G., Fanos V. (2019). Sportomics: Metabolomics Applied to Sports. The New Revolution?. Eur. Rev. Med. Pharmacol. Sci..

[18] Long T., Hicks M., Yu H.-C., Biggs W.H., Kirkness E.F., Menni C., Zierer J., Small K.S., Mangino M., Messier H. (2017). Whole-Genome Sequencing Identifies Common-to-Rare Variants Associated with Human Blood Metabolites. Nat. Genet..

[19] Malsagova K.A., Kopylov A.T., Stepanov A.A., Enikeev D.V., Potoldykova N.V., Balakin E.I., Pustovoyt V.I., Kaysheva A.L. (2023). Molecular Profiling of Athletes Performing High-Intensity Exercises in Extreme Environments. Sports.

[20] Nunes M.J., Moura J.J.G., Noronha J.P., Branco L.C., Samhan-Arias A., Sousa J.P., Rouco C., Cordas C.M. (2022). Evaluation of Sweat-Sampling Procedures for Human Stress-Biomarker Detection. Analytica.

[21] Katsani K.R., Sakellari D. (2019). Saliva Proteomics Updates in Biomedicine. J. Biol. Res.-Thessalon..

[22] Kaur G., Poljak A., Ali S.A., Zhong L., Raftery M.J., Sachdev P. (2021). Extending the Depth of Human Plasma Proteome Coverage Using Simple Fractionation Techniques. J. Proteome Res..

[23] Tanaka M., Wang G., Pitsiladis Y.P. (2016). Advancing Sports and Exercise Genomics: Moving from Hypothesis-Driven Single Study Approaches to Large Multi-Omics Collaborative Science. Physiol. Genom..

[24] Wang M., Yu G., Ressom H.W. (2016). Integrative Analysis of Proteomic, Glycomic, and Metabolomic Data for Biomarker Discovery. IEEE J. Biomed. Health Inform..

[25] Al-Khelaifi F., Abraham D., Diboun I., Elrayess M.A. (2019). Proteomics and Metabolomics Research in Exercise and Sport. Sports, Exercise, and Nutritional Genomics.

[26] Morin J.-B., Samozino P. (2016). Interpreting Power-Force-Velocity Profiles for Individualized and Specific Training. Int. J. Sports Physiol. Perform..

[27] Sanford J.A., Nogiec C.D., Lindholm M.E., Adkins J.N., Amar D., Dasari S., Drugan J.K., Fernández F.M., Radom-Aizik S., Schenk S. (2020). Molecular Transducers of Physical Activity Consortium (MoTrPAC): Mapping the Dynamic Responses to Exercise. Cell.

[28] Platonov V.N. (2005). The System of Athletes Training in the Olympic Sport. The General Theory and Its Practical Applications: Handbook for Trainer of Highest Qualification.

[29] Millet G.P., Roels B., Schmitt L., Woorons X., Richalet J.P. (2010). Combining Hypoxic Methods for Peak Performance. Sports Med. Auckl. NZ.

[30] Millet G.P., Debevec T., Brocherie F., Malatesta D., Girard O. (2016). Therapeutic Use of Exercising in Hypoxia: Promises and Limitations. Front. Physiol..

[31] Stepanov A.A., Malsagova K.A., Kopylov A.T., Rudnev V.R., Karateev D.E., Markelova E.I., Luchikhina E.L., Borisova E.E., Kaysheva A.L. (2023). Determination of Heterogeneous Proteomic and Metabolomic Response in Anti-TNF and Anti-IL-6 Treatment of Patients with Rheumatoid Arthritis. Life.

[32] Conway J., Lex A., Gehlenborg N. (2017). UpSetR: An R Package for the Visualization of Intersecting Sets and Their Properties. Bioinforma. Oxf. Engl..

[33] Sinex J.A., Chapman R.F. (2015). Hypoxic Training Methods for Improving Endurance Exercise Performance. J. Sport Health Sci..

[34] Vellers H.L., Kleeberger S.R., Lightfoot J.T. (2018). Inter-Individual Variation in Adaptations to Endurance and Resistance Exercise Training: Genetic Approaches towards Understanding a Complex Phenotype. Mamm. Genome.

[35] Martin D.S., Levett D.Z.H., Grocott M.P.W., Montgomery H.E. (2010). Variation in Human Performance in the Hypoxic Mountain Environment. Exp. Physiol..

[36] Prior S.J., Hagberg J.M., Phares D.A., Brown M.D., Fairfull L., Ferrell R.E., Roth S.M. (2003). Sequence Variation in Hypoxia-Inducible Factor 1alpha (HIF1A): Association with Maximal Oxygen Consumption. Physiol. Genom..

[37] Delves P.J., Martin S.J., Burton D.R., Roitt I.M. Roitt’s Essential Immunology.

[38] Nieman D.C., Nehlsen-Cannarella S.L. (1991). The Effects of Acute and Chronic Exercise of Immunoglobulins. Sports Med. Auckl. NZ.

[39] Nehlsen-Cannarella S.L., Nieman D.C., Jessen J., Chang L., Gusewitch G., Blix G.G., Ashley E. (1991). The Effects of Acute Moderate Exercise on Lymphocyte Function and Serum Immunoglobulin Levels. Int. J. Sports Med..

[40] Petibois C., Cazorla G., Déléris G. (2003). The Biological and Metabolic Adaptations to 12 Months Training in Elite Rowers. Int. J. Sports Med..

[41] Mackinnon L.T., Hooper S. (1994). Mucosal (Secretory) Immune System Responses to Exercise of Varying Intensity and during Overtraining. Int. J. Sports Med..

[42] Mashiko T., Umeda T., Nakaji S., Sugawara K. (2004). Effects of Exercise on the Physical Condition of College Rugby Players during Summer Training Camp. Br. J. Sports Med..

[43] Nieman D., Tan S., Lee J., Berk L. (1989). Complement and Immunoglobulin Levels in Athletes and Sedentary Controls. Int. J. Sports Med..

[44] McKune A.J., Smith L.L., Semple S.J., Wadee A.A. (2005). Influence of Ultra-Endurance Exercise on Immunoglobulin Isotypes and Subclasses. Br. J. Sports Med..

[45] Mackinnon, Laurel T. (1999). Advances in Exercise Immunology.

[46] Kozlov V.A., Kudaeva O.T. (2002). Immunity and Exercise Stress. Med. Immunol..

[47] Hoffman-Goetz L., Pedersen B.K. (1994). Exercise and the Immune System: A Model of the Stress Response?. Immunol. Today.

[48] Bousquet J., Chanez P., Mercier J., Prefaut C. (1996). Monocytes, Exercise and the Inflammatory Response. Exerc. Immunol. Rev..

[49] Borges G.F., Rama L.M.P.L., Pedreiro S., Rosado F., Alves F., Santos A.M.C., Paiva A., Teixeira A.M. (2012). Haematological Changes in Elite Kayakers during a Training Season. Appl. Physiol. Nutr. Metab. Physiol. Appliguee Nutr. Metab..

[50] I San-Millán I. (2019). Blood Biomarkers in Sports Medicine and Performance and the Future of Metabolomics. Methods in Molecular Biology.

[51] Eleftheriadis T., Liakopoulos V., Antoniadi G., Kartsios C., Stefanidis I. (2009). The Role of Hepcidin in Iron Homeostasis and Anemia in Hemodialysis Patients. Semin. Dial..

[52] Son H.J., Lee Y.H., Chae J.H., Kim C.K. (2015). Creatine Kinase Isoenzyme Activity during and after an Ultra-Distance (200 Km) Run. Biol. Sport.

[53] Brancaccio P., Maffulli N., Limongelli F.M. (2007). Creatine Kinase Monitoring in Sport Medicine. Br. Med. Bull..

[54] Totsuka M., Nakaji S., Suzuki K., Sugawara K., Sato K. (2002). Break Point of Serum Creatine Kinase Release after Endurance Exercise. J. Appl. Physiol..

[55] Clarkson P.M., Kearns A.K., Rouzier P., Rubin R., Thompson P.D. (2006). Serum Creatine Kinase Levels and Renal Function Measures in Exertional Muscle Damage. Med. Sci. Sports Exerc..

[56] Ascensão A., Rebelo A., Oliveira E., Marques F., Pereira L., Magalhães J. (2008). Biochemical Impact of a Soccer Match—Analysis of Oxidative Stress and Muscle Damage Markers throughout Recovery. Clin. Biochem..

[57] Lovell R., Whalan M., Marshall P.W.M., Sampson J.A., Siegler J.C., Buchheit M. (2018). Scheduling of Eccentric Lower Limb Injury Prevention Exercises during the Soccer Micro-cycle: Which Day of the Week?. Scand. J. Med. Sci. Sports.

[58] Russell M., Northeast J., Atkinson G., Shearer D.A., Sparkes W., Cook C.J., Kilduff L.P. (2015). Between-Match Variability of Peak Power Output and Creatine Kinase Responses to Soccer Match-Play. J. Strength Cond. Res..

[59] Silva J.R., Rumpf M.C., Hertzog M., Castagna C., Farooq A., Girard O., Hader K. (2018). Acute and Residual Soccer Match-Related Fatigue: A Systematic Review and Meta-Analysis. Sports Med..

[60] Bonilla D.A., Pérez-Idárraga A., Odriozola-Martínez A., Kreider R.B. (2020). The 4R’s Framework of Nutritional Strategies for Post-Exercise Recovery: A Review with Emphasis on New Generation of Carbohydrates. Int. J. Environ. Res. Public. Health.

[61] Lazarim F.L., Antunes-Neto J.M.F., da Silva F.O.C., Nunes L.A.S., Bassini-Cameron A., Cameron L.-C., Alves A.A., Brenzikofer R., de Macedo D.V. (2009). The Upper Values of Plasma Creatine Kinase of Professional Soccer Players during the Brazilian National Championship. J. Sci. Med. Sport.

[62] Ceciliani F., Giordano A., Spagnolo V. (2002). The Systemic Reaction during Inflammation: The Acute-Phase Proteins. Protein Pept. Lett..

[63] Moshage H. (1997). Cytokines and the Hepatic Acute Phase Response. J. Pathol..

[64] Ogawa K., Sanada K., Machida S., Okutsu M., Suzuki K. (2010). Resistance Exercise Training-Induced Muscle Hypertrophy Was Associated with Reduction of Inflammatory Markers in Elderly Women. Mediators Inflamm..

[65] Yerbury J.J., Rybchyn M.S., Easterbrook-Smith S.B., Henriques C., Wilson M.R. (2005). The Acute Phase Protein Haptoglobin Is a Mammalian Extracellular Chaperone with an Action Similar to Clusterin. Biochemistry.

[66] Bernard D., Christophe A., Delanghe J., Langlois M., De Buyzere M., Comhaire F. (2003). The Effect of Supplementation with an Antioxidant Preparation on LDL-Oxidation Is Determined by Haptoglobin Polymorphism. Redox Rep. Commun. Free Radic. Res..

[67] Spitler D.L., Alexander W.C., Hoffler G.W., Doerr D.F., Buchanan P. (1984). Haptoglobin and Serum Enzymatic Response to Maximal Exercise in Relation to Physical Fitness. Med. Sci. Sports Exerc..

[68] Chen C.-Y., Hsieh W.-L., Lin P.-J., Chen Y.-L., Mao S.J.T., Veas F. (2011). Haptoglobin Is an Exercise-Responsive Acute-Phase Protein. Acute Phase Proteins—Regulation and Functions of Acute Phase Proteins.

[69] Nagashima K., Cline G.W., Mack G.W., Shulman G.I., Nadel E.R. (2000). Intense Exercise Stimulates Albumin Synthesis in the Upright Posture. J. Appl. Physiol. Bethesda Md 1985.

[70] Lee E.C., Fragala M.S., Kavouras S.A., Queen R.M., Pryor J.L., Casa D.J. (2017). Biomarkers in Sports and Exercise: Tracking Health, Performance, and Recovery in Athletes. J. Strength Cond. Res..

[71] Santos R., Tufik S., De Mello M. (2007). Exercise, Sleep and Cytokines: Is There a Relation?. Sleep Med. Rev..

[72] Kraus R.M., Stallings H.W., Yeager R.C., Gavin T.P. (2004). Circulating Plasma VEGF Response to Exercise in Sedentary and Endurance-Trained Men. J. Appl. Physiol..

[73] Donaldson S.K.B., Hermansen L., Bolles L. (1978). Differential, Direct Effects of H+ on Ca^2+^ -Activated Force of Skinned Fibers from the Soleus, Cardiac and Adductor Magnus Muscles of Rabbits. Pflugers Arch..

[74] Sahlin K. (1978). Intracellular pH and Energy Metabolism in Skeletal Muscle of Man. With Special Reference to Exercise. Acta Paediatr. Scand. Suppl..

[75] Bredle D.L., Stager J.M., Brechue W.F., Farber M.O. (1988). Phosphate Supplementation, Cardiovascular Function, and Exercise Performance in Humans. J. Appl. Physiol..

[76] Dale G., Fleetwood J.A., Weddell A., Ellis R.D., Sainsbury J.R. (1987). Fitness, Unfitness, and Phosphate. BMJ.

[77] Nebl J., Drabert K., Haufe S., Wasserfurth P., Eigendorf J., Tegtbur U., Hahn A., Tsikas D. (2019). Exercise-Induced Oxidative Stress, Nitric Oxide and Plasma Amino Acid Profile in Recreational Runners with Vegetarian and Non-Vegetarian Dietary Patterns. Nutrients.

[78] San-Millán I., Stefanoni D., Martinez J.L., Hansen K.C., D’Alessandro A., Nemkov T. (2020). Metabolomics of Endurance Capacity in World Tour Professional Cyclists. Front. Physiol..

[79] Wagenmakers A.J. (1998). Muscle Amino Acid Metabolism at Rest and during Exercise: Role in Human Physiology and Metabolism. Exerc. Sport Sci. Rev..

[80] Shakhmatov I.I., Alekseeva O.V., Kiselev V.I. (2010). The Effect of Training on Reactions of the Hemostatic System under Hypoxic Conditions. Bull. Sib. Med..

[81] Kutafina N.V., Medvedev I.N. (2014). The influence of physical activity on the hemostatic system. Bull. Surgut State Pedagog. Univ..

[82] Bakhareva A.S., Isaev A.P., Savinykh E.Y., Baimukhametov E.F. (2016). Physiological adaptation to huge endurance training loads in athletes. Hum. Sport Med..

[83] Collen D., Semeraro N., Tricot J.P., Vermylen J. (1977). Turnover of Fibrinogen, Plasminogen, and Prothrombin during Exercise in Man. J. Appl. Physiol..

[84] Kutafina N.V., Zavalishina S.Y. (2012). The Mechanisms of Functioning of Vascular–Platelet Hemostasis. RUDN J. Ecol. Life Saf..

[85] Smith J.E. (2003). Effects of Strenuous Exercise on Haemostasis. Br. J. Sports Med..

[86] Schommer K., Menold E., Subudhi A.W., Bärtsch P. (2012). Health Risk for Athletes at Moderate Altitude and Normobaric Hypoxia. Br. J. Sports Med..

[87] Knowles R., Keeping H., Graeber T., Nguyen K., Garner C., D’Amico R., Simms H.H. (1997). Cytokine Control of PMN Phagocytosis: Regulatory Effects of Hypoxemia and Hypoxemia-Reoxygenation. Am. J. Physiol..

[88] Ameln H., Gustafsson T., Sundberg C.J., Okamoto K., Jansson E., Poellinger L., Makino Y. (2005). Physiological Activation of Hypoxia Inducible Factor-1 in Human Skeletal Muscle. FASEB J. Off. Publ. Fed. Am. Soc. Exp. Biol..

[89] Flockhart M., Nilsson L.C., Tais S., Ekblom B., Apró W., Larsen F.J. (2021). Excessive Exercise Training Causes Mitochondrial Functional Impairment and Decreases Glucose Tolerance in Healthy Volunteers. Cell Metab..

[90] Van Gool A., Corrales F., Čolović M., Krstić D., Oliver-Martos B., Martínez-Cáceres E., Jakasa I., Gajski G., Brun V., Kyriacou K. (2020). Analytical Techniques for Multiplex Analysis of Protein Biomarkers. Expert Rev. Proteom..

[91] Heaney L.M., Deighton K., Suzuki T. (2019). Non-Targeted Metabolomics in Sport and Exercise Science. J. Sports Sci..

[92] Saunders P.U., Garvican-Lewis L.A., Schmidt W.F., Gore C.J. (2013). Relationship between Changes in Haemoglobin Mass and Maximal Oxygen Uptake after Hypoxic Exposure. Br. J. Sports Med..

[93] Furrer R., Hawley J.A., Handschin C. (2023). The Molecular Athlete: Exercise Physiology from Mechanisms to Medals. Physiol. Rev..

