# Supplementary material for: Metabolomic and Proteomic Profiling of Athletes Performing Physical Activity under Hypoxic Conditions
Source: Sports (Basel). 2024 Mar 5;12(3):72. doi: 10.3390/sports12030072 (PMC10975304; doi:10.3390/sports12030072)
Supplement: Supplementary file 1 [file sports-12-00072-s001.zip › sports-2796040-supplementary.pdf]

ELISA

Determining the concentration of erythropoietin, interleikin-18 vascular endothelial growth factor, alfa-interferon and tumor necrosis factor were assessed by "sandwich" - option solid-phase enzyme-linked mimmunosorbent assay (ELISA) using a commercial reagent kits («Vector-Best », Russia).

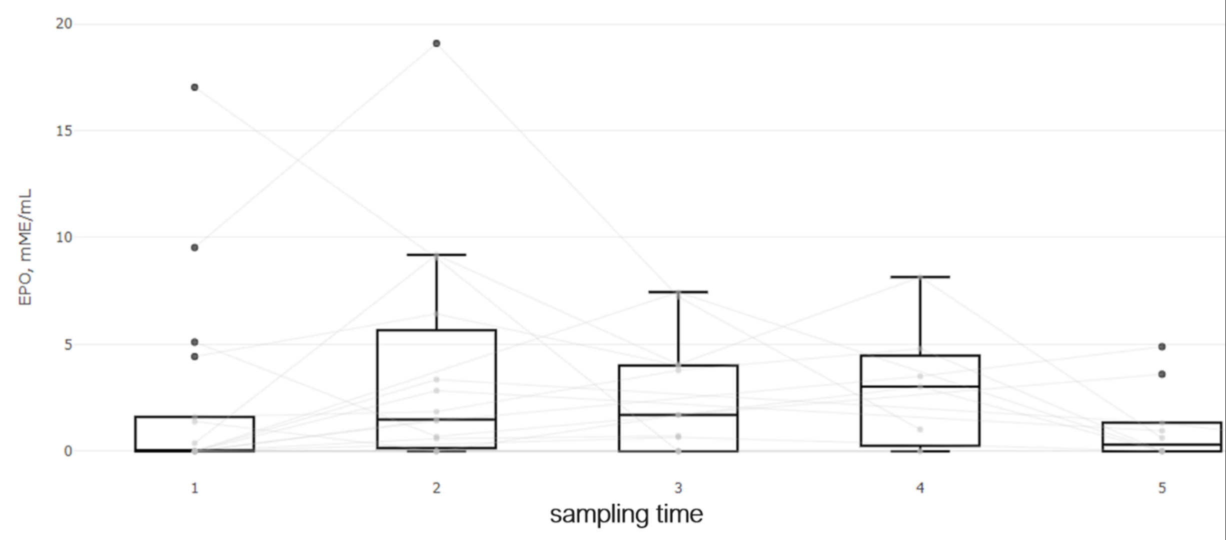

(a)

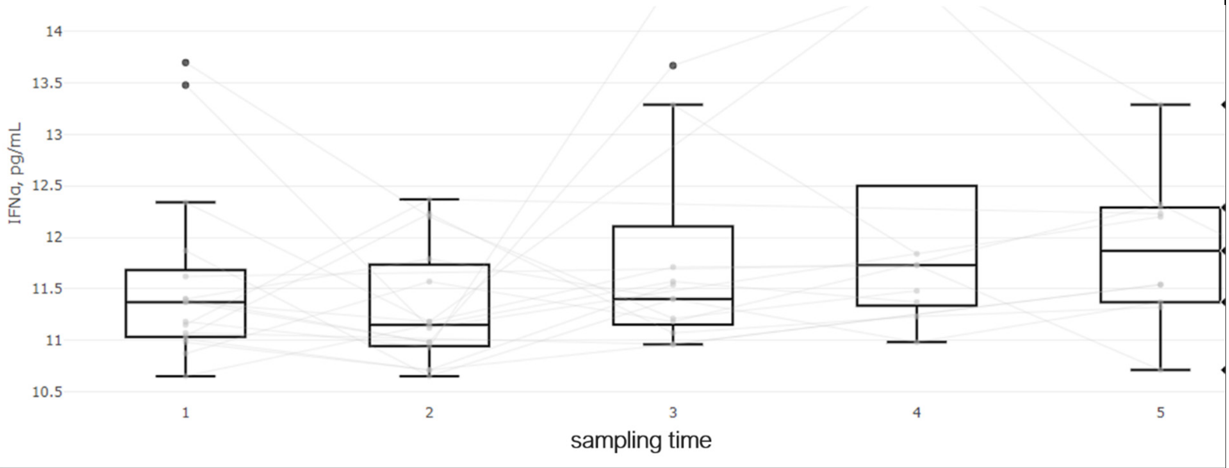

(b)

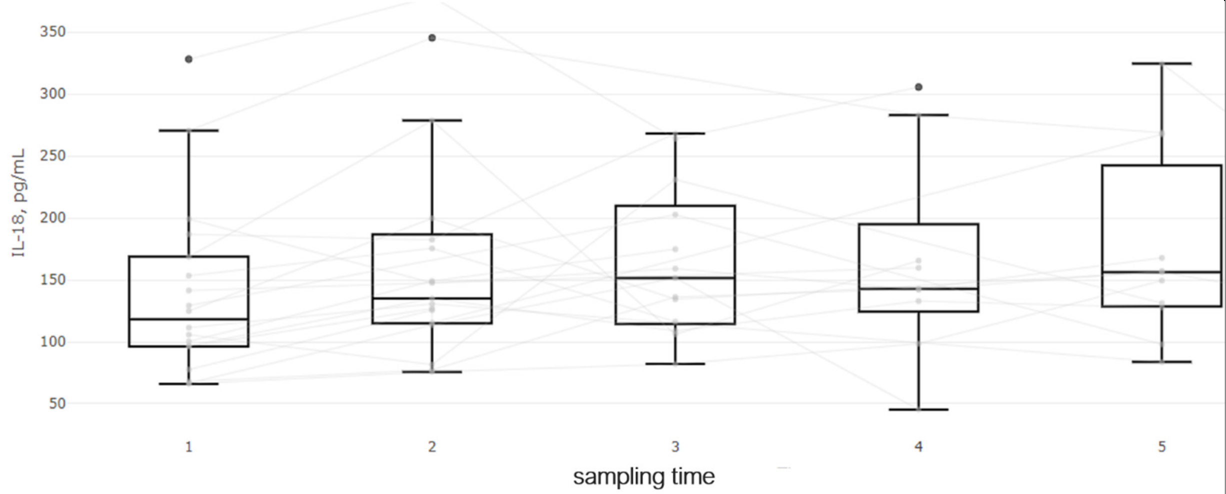

(c)

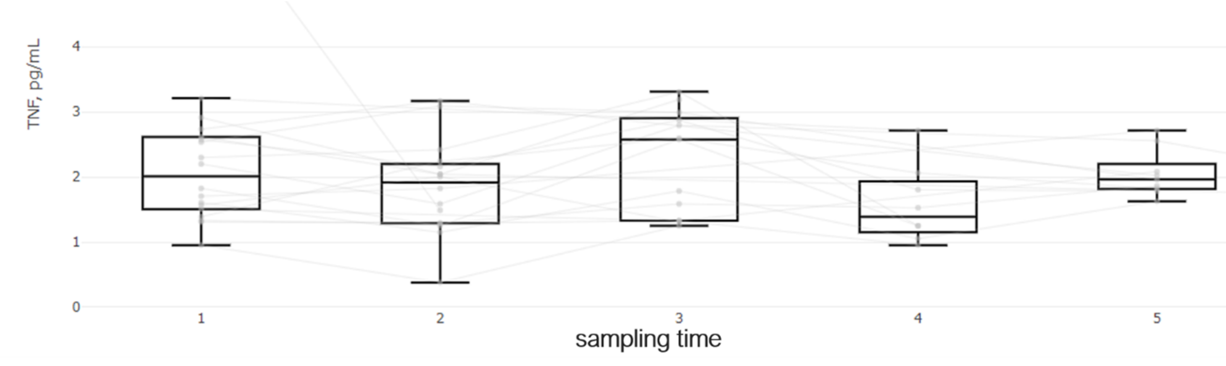

(d)

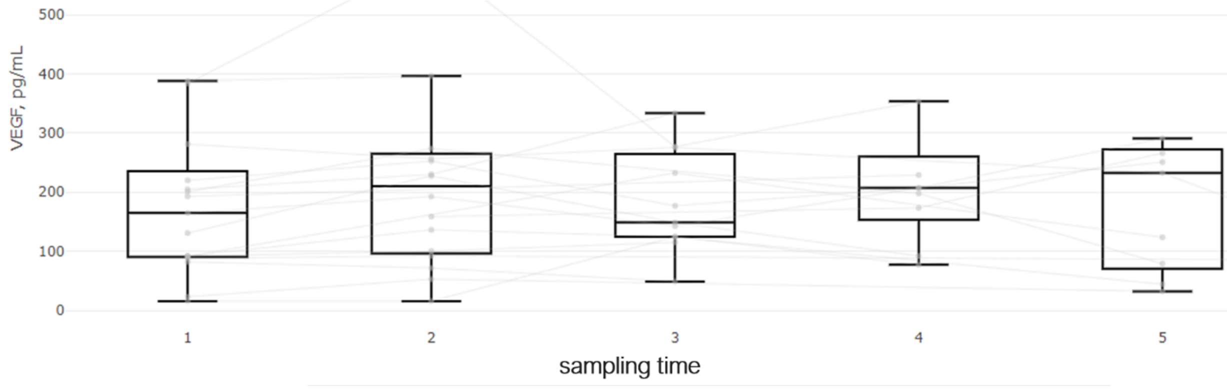

(e)

Figure S1. Results of quantitative determination of the content of erythropoietin (a), alpha-interferon (b), interleikin-18 (c), tumor necrosis factor (d) and vascular endothelial growth factor (e) in the blood plasma of athletes.
